# A Novel Perfused Full‐Thickness Human Skin Microphysiological System for Modeling Injury, Regeneration, Tumor Pathophysiology, and Therapeutics Development

**DOI:** 10.1002/adhm.71445

**Published:** 2026-07-23

**Authors:** Yusuf Surucu, Hamid Malekzadeh, Alexa Rivera Del Rio Hernandez, Naresh Mahajan, Ethan Banks, Dayyan Ur Rahman, José A. Arellano, Katherine S. Yang, Alexey Altman, G.V. Naveen Kumar, Matthew L. Steinhauser, Jeffrey A. Gusenoff, J. Peter Rubin, Asim Ejaz

**Affiliations:** ^1^ Department of Plastic Surgery University of Pittsburgh Pittsburgh Pennsylvania USA; ^2^ Aging Institute of UPMC University of Pittsburgh School of Medicine Pittsburgh Pennsylvania USA

**Keywords:** adipose tissue, bioreactor, ex vivo, human skin, medicine, pathology, perfusion, stromal vascular fraction, taxonomy, New Alternative Method (NAM), Skin

## Abstract

Translating preclinical research findings to clinical applications presents challenges due to the metabolic and anatomical disparities between animal models and humans. Although organ‐on‐a‐chip models replicate the organization of human cells, they lack the complexity of the microenvironment and anatomical fidelity. Developing alternative research models with greater physiological and anatomical relevance to humans holds the potential to enhance translational success and reduce reliance on animal experimentation. In this study, we have showcased the technical details and conditions to maintain the viability of ex vivo perfused large human abdominal fasciocutaneous flaps for up to three weeks. Our efforts involved refining surgical procedures, vascular territory mapping (angiosome analysis), and pedicle exploration; engineering the bioreactor system; and optimizing perfusion media. Angiography via thermal and fluorescent methods was used to confirm the perfusion success. Metabolic activity was closely monitored by tracking glucose consumption and lactate production. Assessments of tissue viability encompassed histological analysis, TUNEL staining, gene expression profiling, measurement of vascular and metabolic reactivity, and in vitro propagation of isolated adipose stem cells and dermal fibroblasts. Furthermore, we harnessed our optimized human skin perfusion model to investigate the dynamics of radiation and chemical‐induced injuries. Additionally, we explored the model's utility in studying adipose tissue metabolism and employed human skin tissue to establish melanoma and breast cancer tumor models. To our knowledge, this is the first model capable of preserving the function and viability of a large flap for extended periods, providing a proof‐of‐concept foundation for diverse research applications.

## Graphical abstract

What if human skin could remain fully alive and functional outside the body for weeks? A New Alternative Method (NAM) based on a vascularized full‐thickness skin microphysiological system achieves precisely this, preserving native vasculature, resident immunity, and metabolic activity for up to three weeks, while faithfully recapitulating radiation injury, tumor invasion, and adipose biology in living human tissue.

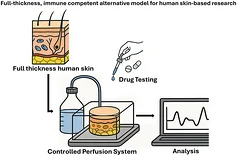

## 1 Introduction

The skin functions as a crucial barrier, safeguarding the body against physical, chemical, and pathogenic threats, while also regulating the bidirectional transport of water, ions, and nutrients [1]. To enhance our understanding of skin structure, function, and related diseases, animal experiments are frequently employed. However, the interpretability of data derived from these experiments to humans may be compromised due to anatomical and interspecies variations, hindering the direct translation of findings to clinical scenarios [1]. In the realm of in vivo models, diverse approaches have been developed for both large and small animals, as well as for humans. Over the past two decades, mouse, rat, and pig models have emerged as predominant choices. The critical consideration of data translation from animals to humans remains paramount [2]. Despite mice being the most commonly utilized laboratory animals, murine skin exhibits differences such as increased skin appendages, fewer epidermal layers, and a loose connection to the underlying muscle. Consequently, pharmaceutical absorption studies conducted on mouse skin may not consistently predict outcomes on human skin [3]. Porcine models, with their skin morphology, physiology, and wound healing processes more closely resembling those of humans, have gained prominence. However, disparities between porcine models and the human condition may impact the translatability of results [4, 5, 6]. As we delve into research involving the skin, the nuanced variations across species underscore the importance of cautious interpretation and consideration of potential limitations in extrapolating findings to human contexts.

Ensuring the translatability of results and minimizing animal experimentation in the preclinical evaluation of novel therapeutic approaches can be achieved through the in vitro modeling of human skin. The global acceptance of the “3Rs” principle replacement, reduction, and refinement, in humane animal research is reflected in numerous national and international legislations [7]. This principle advocates for alternatives to animal testing whenever possible, emphasizing the ethical treatment of animals in research. A significant stride in aligning with the 3Rs principle is evident in the FDA Modernization Act 2.0, signed by the President in 2022 [8]. This act marks a departure from the original Food, Drug, and Cosmetic Act of 1938 (FDCA), which previously mandated animal testing for new drugs approved by the FDA. The revised legislation underscores a commitment to advancing ethical practices in drug development, recognizing the need to explore alternative methods to assess drug safety and efficacy. While human skin in vivo remains the gold standard for skin modeling, its practical use is often impeded by ethical considerations, regulatory constraints, limitations in laboratory facilities, and the potential risks associated with the toxic effects of drugs [9]. Consequently, there exists a compelling need for a fully functional, entirely human in vitro model that faithfully reproduces native skin structure and functionality. Such a model would not only address the ethical concerns surrounding animal experimentation but also provide a reliable platform for preclinical testing, aligning with the evolving landscape of drug development and regulatory standards.

Various in vitro models exist for studying skin biology, ranging from simpler 2D models to more intricate 3D constructs [9]. Basic 2D models involve the cultivation of primary keratinocytes, cell lines, or co‐cultures of keratinocytes and fibroblasts [10]. On the other hand, advanced 3D models are created by culturing single or multiple cell types in distinct layers on a polymeric matrix. The composition of this artificial matrix may include collagen, fibrin fibers, alginate, chitosan, or various synthetic polymers [11]. The cell types employed encompass keratinocytes, fibroblasts, melanocytes, adipocytes, and endothelial cells [9]. One noteworthy 3D model is the Reconstructed Human Epidermis (RHE), developed specifically for in vitro skin irritation assays. This model closely resembles the human epidermis providing a valuable tool for assessing the effects of substances on the skin [12]. More recently, advances in bioprinting have enabled the fabrication of 3D‐printed full‐thickness skin constructs that recapitulate multi‐layered epidermal and dermal architecture with improved architectural precision and high reproducibility [13]. While such bioengineered models offer scalability and compositional control, they are assembled from isolated cell populations and therefore lack the native multicellular complexity, intact vascular network, resident immune microenvironment, and subcutaneous adipose compartment that characterize human skin in vivo.

Additionally, ex vivo models using human or animal tissues have been explored. Human skin samples for ex vivo assays can be sourced from plastic surgery, amputations, or cadavers [9]. Perfusion of ex vivo human skin emerges as a promising strategy, surpassing some aspects of in vitro models. It offers the advantages of maintaining the natural skin architecture, retaining representative cell types in their native microenvironment, preserving the intact resident immune network, and potentially generating a circulatory immune network. Furthermore, this method enables nutrient delivery to cells via a natural vascular network, a notable improvement over diffusion in most artificial systems. However, employing ex vivo perfused human tissue as a platform for studying skin pathologies and drug discovery presents challenges. The foremost challenge lies in sustaining the viability of the perfused tissue over an extended period. Previous studies indicate that tissue viability can be maintained for 6‐8 h using artificial perfusion fluids or for up to 4 days using whole‐blood perfusion [14, 15]. Overcoming this hurdle is crucial to unlocking the full potential of ex vivo perfused human tissue for advancing our understanding of skin biology and facilitating drug discovery efforts.

In summary, existing skin model systems each carry distinct limitations that constrain their translational utility. Murine skin models differ from humans in epidermal thickness, hair follicle density, and wound healing kinetics, limiting direct extrapolation of drug and injury responses [3]. Porcine models, while anatomically closer to humans, carry cost, ethical, and interspecies translational limitations [4, 5, 6]. Organotypic 3D skin equivalents composed of keratinocytes and fibroblasts lack vasculature, a resident immune network, and the adipose compartment, and are typically limited to studies of <7 days [9, 10]. Non‐perfused human skin explant models can preserve tissue architecture for 4–7 days at most; beyond this window, progressive anoxia and waste accumulation render the tissue nonviable [16]. Microfluidic skin‐on‐chip devices, while scalable, are assembled from isolated cell populations seeded onto synthetic substrates and cannot recapitulate the full structural complexity of intact human skin [17]. In this study, we describe an ex vivo perfused full‐thickness human skin model that substantially extends tissue viability (sustaining functional tissue for up to three weeks), preserves native vasculature, immune cells, and adipose tissue, and enables multi‐domain applications in a single human‐tissue system. Whether the biological responses observed are exclusively achievable through this perfusion approach or could in principle be recapitulated by future advanced engineered systems remains an open question; our findings establish the platform's capability and provide a basis for such comparative studies.

## 2 Materials and Methods

### 2.1 Tissue Collection

Abdominoplasty tissues were collected from female donors undergoing lipectomy procedures at the University of Pittsburgh Medical Center (UPMC) and the Center for Organ Recovery and Education (CORE) in Pittsburgh. Tissues from nineteen donors aged 38 ± 14 years, with a BMI of 28 ± 4, were used in this study. De‐identified demographic data were recorded for all donors to characterize inter‐donor biological variability across experimental outcomes. To account for donor‐to‐donor heterogeneity, experimental conditions were standardized across tissues with respect to ischemia duration, perfusion parameters, and media composition. The procedure of tissue collection was approved by the University of Pittsburgh Institutional Review Board (IRB No. 0511186).

### 2.2 Pedicle Isolation and Cannulation

Tissue specimens were transported to the laboratory in sterile containers immediately after collection. Surgical waste tissue is placed into a sterile bag, which is then closed and placed inside a closed polypropylene bucket. The bucket is kept in an insulated box to maintain ambient temperature while it is transported to the laboratory facilities with measures to ensure tissue sterility. All subsequent procedures were conducted within BSL2 biosafety hoods to maintain aseptic conditions and due to the use of potentially toxic chemicals for experimentation (Nitrogen Mustard) as per the EH&S guidelines at the University of Pittsburgh. To ensure complete sterility, all equipment, including the bioreactor and dissection microscopes, underwent sterilization using ethylene oxide (EtO) treatment. The isolation of the deep inferior epigastric perforators or superficial inferior epigastric system pedicles was carried out by a skilled microsurgeon. The diameter of the pedicle was accurately measured with ophthalmic calipers, and suitable, flexible Teflon cannulas were selected and placed for the artery (ranging from 20 to 26 G) and the vein (ranging from 18 to 22 G). To maintain the integrity of the flaps, a thorough flush was performed using 250 mL of heparinized saline solution. The procedure included constant monitoring of pressure levels, ensuring it remained below 140 mmHg during the flushing process.

### 2.3 Angiography and Infrared Imaging

Fluorescein saline (1%) solution was introduced and circulated through the system, with the utilization of a UV lamp (Wood's lamp) to visually track and assess the perfusion area both before and after bioreactor‐based perfusion. Additionally, an infrared camera (Flir) is employed to conduct thermal imaging of the flap's perfusion dynamics throughout the entire perfusion process.

### 2.4 Bioreactor System Operation

The pannus tissue was carefully positioned on a specially designed grill structure, which was supported by a sterile plastic platform to prevent any undue pressure and injury to the skin. This entire tissue assembly was then elevated above a collection tray. Subsequently, the assembly was moved into an enclosed chamber with a relatively small opening for the necessary tubes and cables. The chamber itself was equipped with a continuous heating system facilitated by a controlled heating pad device. It was filled with sterile water, and the water was refreshed as needed to maintain an optimal level of humidity. A thermal sensor probe was inserted into the tissue to enable real‐time monitoring and recording of the tissue's temperature. The artery, which had been cannulated, was connected to the bioreactor system through sterile tubing. A closed circulatory system was established, with the perfusion media being recirculated from the vein and the collection tray back to the reservoir. The bioreactor system was composed of several key components, including a peristaltic pump, a narrow‐mouthed media reservoir, a gas exchanger, a hemofilter, pressure monitors, an in‐house built bubble trap, a manometer, an in‐house built in‐line media heater and chamber heater, Masterflex pump tubing, and perfusate thermal sensors. This entire perfusion operation was designed to be closely monitored, controlled, and recorded via integrated Logitech cameras and remote access to the pumps, heaters, and gas exchange through individualized software. The perfusion media followed a sequence within the system, passing through various components: 1) a gas exchanger for pH balancing and oxygenation, 2) an automated pressure sensor for continuous data logging, 3) a bubble trap to prevent any air from reaching the tissue through the tubing, 4) a manual manometer for pressure sensor calibration and validation, 5) a media heater for warming the perfusate, 6) a flow sensor featuring an integrated thermometer for recording flow rate and temperature, and 7) in line pressure sensor.

### 2.5 Perfusion Media

The skin flap was perfused using high‐glucose DMEM, supplemented with 1% antibiotic/antimycotic solution and 5 g/dL of bovine serum albumin. The perfusion media is composed of the following DMEM/High Glucose, penicillin‐streptomycin solution (1%), amphotericin B (0.5%), gentamicin (0.1%), HEPES solution (10 mM), bovine serum albumin (5 g/dL), hydrocortisone solution (25 mg/L), fluconazol (500 mg/mL) and ciprofloxacin (1 mL/l).

The media was sterilized by passing it through 0.2 µm filter. The media was circulated throughout the system, which had a total volume of two liters, and was refreshed every third day during the ex vivo perfusion to ensure its continued efficacy. Our media is prewarmed at 37°C before running the perfusion system, and the heating device helps to maintain this temperature in the circulating media that goes straight to the tissue. This allowed us to maintain the appropriate conditions for our research objectives and the viability of the tissue during the study.

### 2.6 Perfusion Timeline

Upon arrival at the laboratory, the tissue samples were promptly assessed. The weight of the tissue was measured, and detailed photographs were taken to document their gross morphology. Following this initial evaluation, the candidate hemi‐panni flaps with robust pedicles were carefully selected for further processing, which involved skeletonization and cannulation. These selected flaps then underwent a thorough heparin (2000 Units) wash, followed by angiography to assess vascular integrity, and were subsequently connected to the perfusion system. This entire process, from tissue arrival to the initiation of perfusion, typically spanned approximately 2‐3 h, resulting in an acceptably brief cold ischemia time of 3‐4 h, which is well within the acceptable range for such procedures. As part of our standard protocol, data logging commenced concurrently with the start of perfusion. Throughout the study, daily samples were methodically collected for various analyses, including histological evaluation, monitoring of glucose and lactate levels, assessment of pH and electrolyte concentrations, and measurements of adiponectin and soluble gas levels in the perfusion media. To quantify glucose and lactate metabolism, a standardized formula was employed, calculating these values based on changes in concentration in the media, the total volume of media, the duration of change (in hours), and the total weight of the flap.

$$\mathrm{Metabolism}=\frac{\Delta \;\mathrm{Concentration}\;\mathrm{in}\;\mathrm{media}\;\times \mathrm{Total}\;\mathrm{volume}\;\mathrm{of}\;\mathrm{media}}{\Delta \mathrm{hours}\;\times \mathrm{Flap}\;\mathrm{total}\;\mathrm{weight}}$$

This comprehensive monitoring and data collection process ensured the accuracy and reliability of our results.

### 2.7 Histology

Skin biopsies were collected in a 4% paraformaldehyde solution. Following a standard protocol, the samples were processed for paraffin embedding. Sections, 5 µm thick, were then cut using a Leica RM 2255 microtome. These sections were stained with Hematoxylin and Eosin (H&E) and Masson's trichrome stain for histological analysis [18]. Images were captured using a Keyence microscope in bright‐field mode at magnifications of 20×, 100×, and 200×.

### 2.8 Immunofluorescence Staining

Punch biopsies were preserved with 4% PFA (paraformaldehyde), and sections were stained for specific antigens [18]. TUNEL staining was performed following the kit protocol (Sigma, USA). Sections were stained using antibodies for Perilipin, Sox 10, Ki‐67, β‐actin, PARP (Proteintech, USA), and DAPI Stain (Sigma, USA).

### 2.9 Quantitative RT‐PCR Gene Expression

Total RNA was extracted using the RNeasy Micro Kit (Qiagen, Hilden, Germany), and cDNA synthesis was conducted using the RevertAid First Strand cDNA Synthesis Kit (Thermo Fisher Scientific, Waltham, MA, USA). Gene‐specific primers were acquired from Sigma‐Aldrich, St.

Louis, MO, USA. For quantitative expression analysis, Quantstudio 3 (Thermo Fisher Scientific, Waltham, MA, USA) was employed. To normalize the mRNA quantification, β‐actin was used as the reference gene. The data for each gene transcript were normalized by calculating the difference (∆Ct) between the Ct values of the housekeeping gene and the target gene. The relative increase or decrease in expression was determined by comparing the reference gene to the target gene using the ∆∆Ct method. The relative expression was calculated using the formula (= 2∆∆Ct).

### 2.10 RNA Sequencing

The study comprised two groups: a control group (non‐irradiated) and an irradiated group exposed to a single dose of 15 Gy x‐ray radiation. Skin biopsies were collected from both groups under sterile conditions and immediately snap‐frozen in liquid nitrogen. Samples were subsequently stored at −80°C until further processing to preserve RNA integrity and prevent degradation. Total RNA was isolated from frozen tissue biopsies using the Qiagen RNeasy Fibrous Tissue Mini Kit (USA) according to the manufacturer's protocol. The purity and concentration of extracted RNA were determined spectrophotometrically using NanoDrop (Thermo Fisher Scientific).

Qualified RNA samples from both control and irradiated groups were submitted to Novogene (USA) for bulk RNA sequencing. Library preparation was performed using the Illumina TruSeq RNA Library Prep Kit followed by paired‐end sequencing on the Illumina NovaSeq 6000 platform. The sequencing generated high‐quality raw reads, which were aligned to the human reference genome (GRCh38.p14) using standard alignment pipelines. The resulting expression data were processed as raw count matrices for downstream analysis.

Gene expression analysis was performed using iDEP 96 platform for integrated differential expression and pathway exploration. Normalization and transformation were applied to raw counts prior to principal component analysis (PCA), which was used to assess global transcriptomic variation among samples. PCA revealed a clear separation between the control and irradiated groups, indicating distinct expression profiles. Differentially expressed genes (DEGs) were identified using a threshold of adjusted *p* < 0.05 and log2‐fold change>1. The resulting dataset comprised upregulated, downregulated, and statistically insignificant genes, visualized in a volcano plot to highlight the magnitude and confidence of differential expression. Significant DEGs were analyzed using Ingenuity Pathway Analysis (IPA; Qiagen, Redwood City, CA, USA) to explore biological pathways and molecular mechanisms affected by irradiation. IPA's *Core Analysis* tool was used to map DEGs onto curated molecular networks, predicting pathway activity through z‐score–based enrichment. In the resulting pathway activity plot, orange bars (light to dark) denote activated pathways (positive z‐score), blue bars (light to dark) represent inhibited pathways (negative z‐score), and white bars indicate no change (z‐score = 0). The vertical threshold line represents the statistical significance cutoff (*p* < 0.05), with pathways exceeding this threshold considered significantly enriched.

All data visualization and interpretation were performed using iDEP and IPA platforms. PCA, volcano plots, z‐score‐based pathway enrichment maps, and Gene Ontology (GO) bubble plots were generated to summarize transcriptomic alterations between control and irradiated samples. Integration of these analyses provided a holistic view of radiation‐induced molecular reprogramming, highlighting a coordinated suppression of oxidative stress and inflammatory pathways accompanied by activation of extracellular matrix remodeling, angiogenic, and reparative signaling networks.

### 2.11 Cells Isolation and Propagation

Hemi‐flaps were utilized for perfusion through our system, while contralateral hemi‐flaps were employed to establish two diffusion‐based culture methods to evaluate cell viability. In the first method, one half was positioned in a tray filled with media with its adipose side facing down. The culture media was refreshed every 2 days. This served as the sample's negative control, referred to as the “float” model. The second half of the hemi‐pannus was placed with its adipose surface exposed, and a gauze soaked in media was employed to facilitate nutrient delivery via diffusion. This model was denoted as the “cover” model. On the third day, both dermal and adipose tissues were harvested and processed to isolate and seed dermal fibroblasts and adipose stem cells, following established protocols. After isolation, cell suspensions were assessed for viability using trypan blue staining. The percentage of viable cells was determined and compared between the groups. The seeded cells were then cultured for seven days in T25 cell culture flasks (Thermo Fisher, USA) and examined under a bright‐field microscope at 20x magnification [19, 20].

Subsequently, the culture dishes were treated with 4% paraformaldehyde and stained with β‐actin and DAPI to visualize the cells. The stained dishes were imaged using an immunofluorescence microscope at 200 times magnification.

2.12 Vascular Reactivity Test We employed epinephrine and norepinephrine mix 50 nM (vasoconstrictors) as well as papaverine 10% (vasodilator). Vascular activity assessments were conducted on both day 5 and day 12 of the perfusion experiment. The standard perfusion media was replaced with media containing vasoconstrictors, and this switch was accompanied by continuous monitoring and recording of pressure and flow rate. After a duration of 2 h, the vasoconstrictor‐containing media was subsequently replaced with media containing vasodilators. Throughout this process, pressure and flow rate were continually monitored and recorded.

### 2.12 Skin Tissue Explant Culture Kindly change the numbering of the headers

Explant culture was set up using 8 mm punch biopsies collected from freshly harvested abdominoplasty pannus specimens. Tissue biopsies were cultured in six‐well plates in an air‐liquid interface using *trans*‐well cell culture constructs. DMEM+5% BSA media was used for culturing the explants. Biopsies were fixed in 2% paraformaldehyde for further histological processing.

### 2.13 Flow Cytometry

Cell‐cultured human adipose‐derived stem cells (ASCs) and dermal fibroblasts were trypsinized. Cells were fixed and permeabilized using the Cytofix/Cytoperm kit protocol (BD Biosciences, USA). Cells were stained with CD34‐APC, CD146‐BV421(BD Biosciences, USA), CD90 PE‐Cy7, CD105 PerCpCy5.5, CD24 BV605 (Biolegend, USA), Vimentin (Thermo Fisher, USA), or isotype control. Cells were analyzed using LSRFortessa (BD Biosciences, USA), and data were analyzed using FlowJo software.

### 2.14 Transepidermal Water Loss (TEWL) Measurement

Epidermal barrier integrity was assessed by measuring transepidermal water loss (TEWL) using a GPower TEWL meter (GPOWER Inc., Republic of Korea) at Days 0, 5, and 10 post‐perfusion initiation. Before each measurement, the perfusion chamber was opened, and the tissue surface was gently blotted with sterile gauze to remove excess moisture from the perfusion environment. The tissue was then allowed to equilibrate at room temperature (22°C ± 1°C) with relative humidity maintained at 40%–60% for a minimum of 15 min before measurement. The GPower probe was applied perpendicularly to the skin surface with gentle, consistent contact pressure at a standardized anatomical location within the well‐perfused zone confirmed by thermal imaging. Three replicate measurements were recorded per time point, with a 30‐second stabilization interval between readings. The mean of the three measurements was used for analysis. Results are expressed in g/m^2^/h.

2.16 Insulin Challenge Cannulated tissue was perfused with insulin(30U) ‐containing perfusion media on days 5 and 12. 0 hour media samples were collected and subsequently every hour for 4 hours. Glucose consumption and lactate production were measured with calibrated lactate and glucose strip meters. The rate of metabolism was calculated with the following formula, x = Δ concentration in media * total volume of media / (Δ hours * flap total weight). Metabolic rates were plotted against time points. Insulin challenge analysis was performed on day 5 and day 12 post perfusion.

### 2.15 Lipolysis Analysis

Perfusion media containing epinephrine and norepinephrine (Sigma, USA) was perfused through cannulated tissue. 0 ho samples and subsequent samples were collected every hour and snap‐frozen. Media samples were analyzed for triglycerides (Stanbio), NEFA (Wako), and glycerol (Sigma, USA) contents following protocols from the manufacturer.

### 2.16 Chemical Injury Model‐Nitrogen Mustard Application

Designated areas within the well‐perfused skin region confirmed through angiography were selected for nitrogen mustard exposure or the control treatment. The nitrogen mustard powder (Sigma, USA) was reconstituted in acetone to enhance its penetration into the skin. For the vehicle control, acetone alone was used. The nitrogen mustard solution of 30 mg/cm^2^ was meticulously applied to the skin using a pipette, and a cotton swab saturated with the solution was used to ensure uniform distribution over the marked skin area. Photographs of the treated areas were taken at intervals of 30 min, 6, 24, 120, and 200 h post‐application, followed by punch biopsies collection. On day 12, angiography was performed once more to confirm that the application area still exhibited good perfusion. This step was taken to ensure that any observed changes in the samples resulted exclusively from the application of the agent. Biopsy samples were preserved either through snap freezing and subsequent storage at −80°C or by fixation with 2% paraformaldehyde.

### 2.17 Cancer Modelling

Breast cancer cell lines MCF‐7, MDA‐MB‐231, or melanoma cancer cells OMM 2.3, A375, and MV3 are cultured in high‐glucose DMEM supplemented with 1% antibiotic/antimycotic and10% FBS. Once the cells reached confluency, they were trypsinized and counted before injection. Cells for injection were resuspended in 100 µL perfusion media and delivered into the marked dermis area using a 16 G needle on day 1 post perfusion. 1x10^5^‐1x10^6^ cells per 100 uL per injection site were employed. Tissue biopsies were collected on day 16 post‐injection and fixed in 2% paraformaldehyde solution.

### 2.18 Statistical Analysis

Statistical analyses were performed using GraphPad Prism 10 (GraphPad Software Inc., La Jolla, CA, USA). Differences between group means were evaluated using either Student's t‐test for comparison between two groups or analysis of variance (ANOVA) for multiple comparisons. Error bars in the figures denote the mean ± SD (Standard Deviation), providing a measure of the variability within each dataset. Statistical significance levels were denoted as follows: * *p* < 0.05, with values considered significant at *p* <0.05.

## 3 Results

### 3.1 Superficial Inferior Epigastric Artery (SIEA) and Superficial Inferior Epigastric Vein (SIEV) Are the Optimal Perforators to Perfuse the human Panniculectomy Flap

**FIGURE 1:**
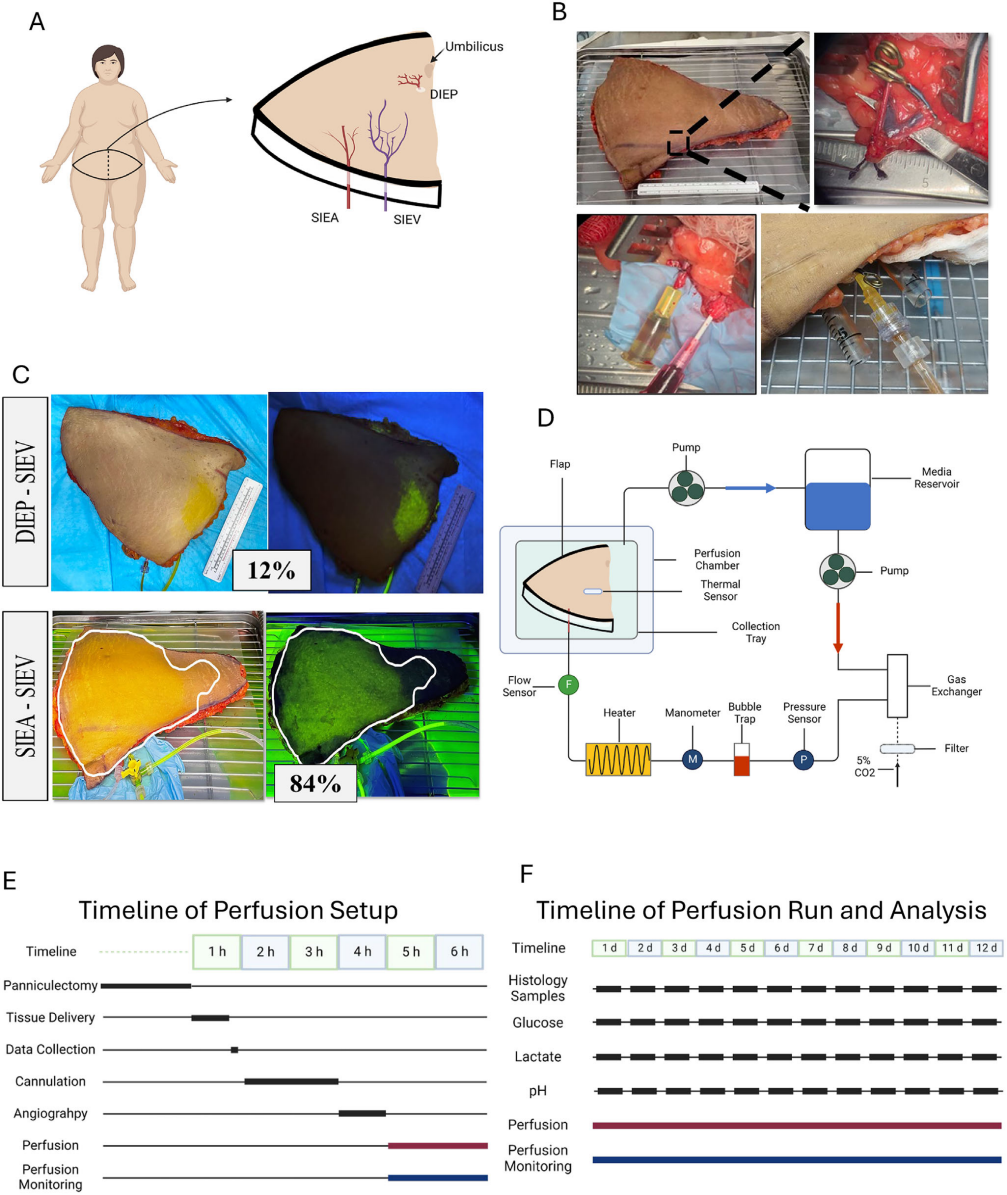
Optimization of vasculature for efficient perfusion. (A) Schematic of major perforator systems in the abdominal faciocutaneous flap. (B) Surgical preparation and cannulation of the pedicle. (C) Fluorescein angiography of perfusion tissue through the DIEP and SIEA systems (*n*=3). (D) Schematic of perfusion bioreactor. (E) Timeline of the perfusion system set‐up. (F) Schematic of the timeline of perfusion run and analysis.

The initial phase of developing a practical and reproducible tissue perfusion system, designed to sustain tissue viability over extended durations and enhance tissue perfusion coverage, commences with a thorough understanding of tissue anatomy and the identification of vascular perforators that supply the tissue. In the context of abdominal panniculectomy flaps, two primary perforators play a crucial role in tissue irrigation: the deep Inferior epigastric perforators (DIEP) and the superficial inferior epigastric artery (SIEA) in conjunction with the superficial inferior epigastric vein (SIEV) (Figure 1A). Meticulous skeletonization and cannulation of these pedicles were performed with the precision of a dissecting microscope within a biological safety cabinet, accurately gauging the pedicle diameter using ophthalmic calipers. For this purpose, we employed flexible Teflon cannulas within the range from 20 to 26 G (Figure 1B). After heparin washing, tissues cannulated through either the DIEP or SIEA/SIEV system underwent Fluorescein angiography (Figure 1C). The assessment of the fluorescing tissue area revealed that SIEA/SIEV perfusion achieved an impressive 84±8% perfused area, whereas DIEP perfusion resulted in a mere 12% ± 3% perfused tissue area (Figure 1C). As most abdominoplasty procedures yield two hemi‐flaps suitable for use as test specimens and control tissues, it is feasible to utilize them in such a manner. Alternatively, cannulation of both the DIEP and SIEA/SIEV systems with an anatomical separation of the cannulated tissue portion can be employed, rendering it equally valuable for diverse downstream applications.

Our dedicated perfusion bioreactor is designed with the physiological needs of skin tissue in consideration and comprises a perfusion chamber along with a collection tray to maintain the tissue in a humid environment and collect media outflow, thereby sustaining a closed circulatory system (Figure 1D). This bioreactor system is equipped to perfuse the tissue with media at a physiological temperature of 33°C–35°C, supplemented with 5% CO_2_, and to maintain a controlled flow rate that keeps the pressure within the range of 45‐65 mm of Hg (Figure 1D). To minimize warm ischemia time, we have established a standardized timeline for pre‐ and post‐perfusion operations, with the routine pre‐perfusion processes, including tissue transportation, data collection, and cannulation, being typically completed within 3.5 h from the commencement of the surgery. Subsequently, angiography and perfusion procedures are carried out (Figure 1E). As part of the regular operational analyses during perfusion, we conducted histological sampling and daily monitoring of glucose, lactate, and pH levels to maintain physiological perfusion conditions (Figures 1F, 2B,C).

FIGURE 2Metabolic and histological analysis of perfused tissue. (A) Thermal imaging of the perfused tissue. Data representative of 15 flaps. (B) Graphical representation of media flow rate, tissue temperature, pressure, and media pH during perfusion runs. (C) Graphical representation of glucose consumption rate and lactate production during skin perfusion over the days (*n*=3). (D) Gross morphology of abdominal skin flaps before and at the end of perfusion run. (E and F) H & E‐stained histology sections of full‐thickness skin before and at different days post perfusion. (E) Scale bar=100 µm (20x objective) and (F) Scale bar=50 µm (40x objective) (*n*=5). (G) Skin sections were stained with antibodies specific to Langerhans (langerin), mast (tryptase), macrophages (CD11b), and dendritic (CD11c) cells. Scale bar=20 µm (20x objective).

**Figure:**
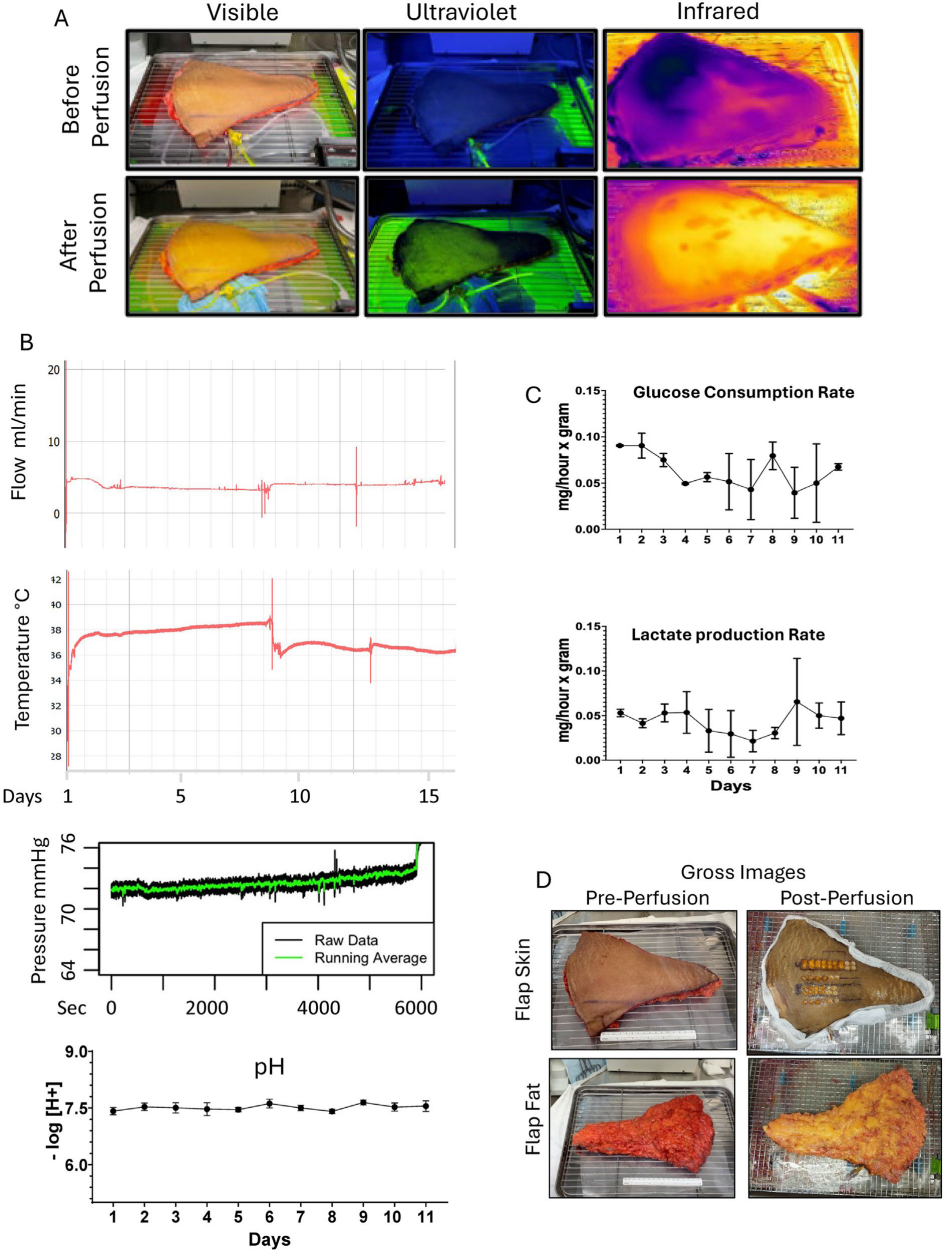

**Figure:**
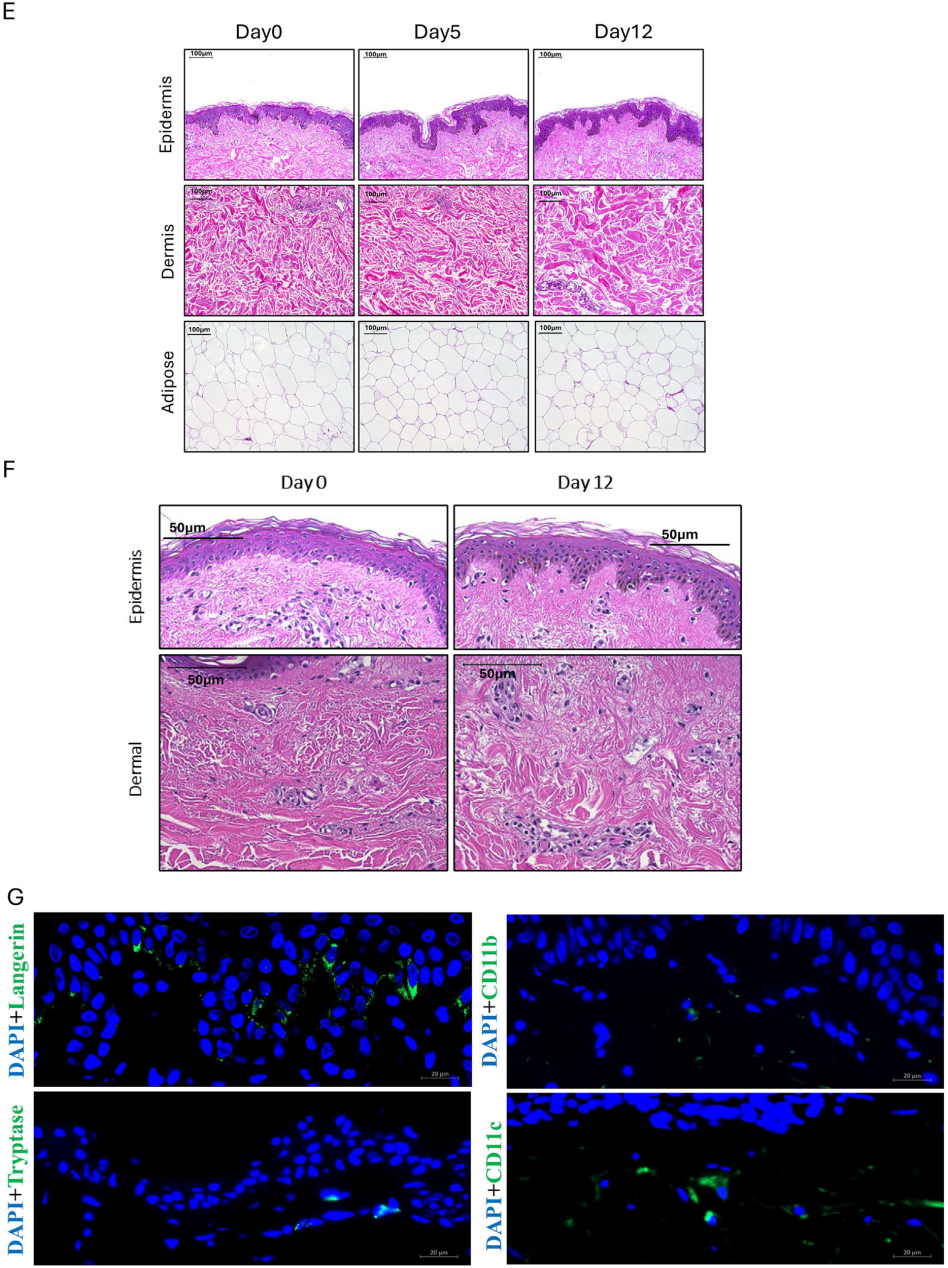

### 3.2 Our Optimized Methodology Maintains Skin Tissue Viability Ex Vivo for up to Three Weeks

In addition to confirming the optimal perfusion of skin tissue through Fluorescein angiography at the onset of the perfusion process, we chose to incorporate thermal imaging of the skin using a handheld infrared camera (Figure 2A). Our all‐encompassing perfusion bioreactor software is adept at the continuous monitoring and recording of key perfusion parameters, including flow rate, media and tissue temperature, pressure, and pH of the perfusion media. Upon cannulation and starting the perfusion through the bioreactor, we started analyzing the metabolic activity dynamics within the skin tissue (Figure 2B). The flow rate and media temperature flowing to the tissue are continuously monitored, and reading patterns up to 15 days are shown in Figure 2B. While pressure readings are also recorded continuously, the readings from the first 100 min are shown (Figure 2B). The system is capable of recording and storing the values from the above‐mentioned parameters up to each second of the perfusion operation, resulting in thousands of data values. Our assessments of glucose consumption and lactate production demonstrated a relatively high rate of glucose consumption and, correspondingly, lactate production by the flap tissues in the initial 3 h following the commencement of perfusion at a relatively consistent pH (Figure 2B,C). Subsequently, we observed a more consistent rate of glucose consumption and lactate production at later time points (Figure 2C). Notably, the tissue maintained a normal gross morphology, with a subsequent reduction in redness attributable to the clearance of red blood cells through cell‐free perfusion media (Figure 2D). Histological examination of the perfused skin sections at various time intervals post‐perfusion, evaluated by multiple dermatopathologists, verified the maintenance of skin architecture for an extended period following perfusion. Notably, there were no discernible changes in the appearance of the epidermis when compared to fresh tissue samples, and both the dermal layer and subdermal adipocyte layer retained their cellular integrity (Figure 2E,F). Histology sections from day 20 post‐perfusion tissue showed retained integrity of epidermal and dermal junction and healthy vascular structure. We observed a few degenerative changes in the cells in the epidermal layer (Figure S1). In addition, immunofluorescence staining confirmed the presence of Langerhans, mast, macrophages, and dendritic cells in the skin section at day 13 post perfusion (Figure 2G).

To further assess the health of the perfused tissue, we stained the skin sections for apoptotic cells using TUNEL stain. Quantification of TUNEL‐positive cells in the skin sections from fresh tissue and tissue perfused for 5 and 12 days revealed a slight increase in TUNEL positivity by day 12, which corresponds to the normal half‐life of keratinocytes (Figure 3A,B). Our control non‐perfused tissue, submerged in media, displayed nearly complete cell death by day 7, substantiating the effectiveness of our perfusion operation in preserving tissue viability (Figure 3C). Non‐perfused tissue showed clear signs of necrosis and decomposition by day 7. Analyzing the genes associated with tissue hypoxia and inflammation unveiled a significant decrease in the expression of HIF 1α, IL‐6, and NF‐κB within the first six hours of perfusion, and these reductions were sustained for up to 264 h of analysis time (Figure 3D). In addition, damage‐associated markers GPX1, HMGB1, IL‐1, TNFα, and TGF‐β showed no significant increase in expression up to 13 days of monitoring post‐perfusion (Figure S2). To further validate the preservation of cell viability in the perfused tissue, we isolated adipose‐derived stem cells (ASCs) and dermal fibroblasts at different time points from the perfused tissue and assessed their viability and proliferation in cell culture. The ASCs phenotype was confirmed by analyzing the positive expression of CD34, CD146, CD90, CD105, and CD24 in these cells by flow cytometric analyses (Figure S3) [21, 22]. The phenotype of isolated dermal fibroblasts was confirmed by positive expression of Vimentin, CD90, and CD34 (Figure S4) [23, 24]. The results demonstrated that the isolated cells exhibited viability comparable to those isolated from fresh tissue, and they displayed robust proliferation in cell culture (Figure 3E,G). In contrast, we observed no viable cells in the non‐perfused control tissues that were either submerged in media or covered with gauze pieces wet with media, under the same conditions in the hood as our perfused tissue (Figure 3F,G). Ex vivo explant biopsy culture is a well‐established, routinely used alternative method for conducting experiments on human skin to study various pathologies and test drug candidates. Employing tissue from the same donor, we compared diffusion‐cultured ex vivo explant biopsies with perfused tissue using our bioreactor‐mediated arterial perfusion approach. Our endpoint analysis focused on tissue integrity measured in terms of maintenance of the epidermal‐dermal junction over time using H&E staining. We observed the start of disintegration of the junction by day 3 in the biopsy culture, and by day 5, the junction was completely disintegrated, while we saw no visible histological changes in perfused tissue even at day 8 (Figure 3H). Perilipin, which coats the surface of lipid droplets is a key marker of adipocyte health and metabolism. Given that we utilized full‐thickness human skin tissue in our perfusion reactions, including the intact subcutaneous adipose tissue layer, we conducted an analysis of perilipin expression in adipocytes at various stages post‐perfusion. The results demonstrated the maintenance of perilipin expression in perfused tissue for up to 12 days, while our control tissue exhibited primarily cell debris by day 3 (Figure 3I,J). Sustaining the function of endothelial cells and vasculature is vital for ensuring the optimal flow of nutrients to the cells. To assess the capability of our perfusion system to maintain normal vasculature function in the perfused tissue, we examined the response of the vasculature to vasoconstrictor and vasodilator agents in the media at different time points post‐perfusion (Figure S5). The measurements revealed an increase in pressure and a decrease in flow rate upon the application of adrenaline (a vasoconstrictor), with the pressure reaching nearly three‐fold within two hours of adrenaline application (Figure 3K). Transitioning to media containing papaverine (a vasodilator) at the peak pressure led to a reduction in pressure and an increase in flow rate within the first hour of application (Figure 3K). In addition, we assessed epidermal barrier integrity of perfused tissue by measuring *trans*‐epidermal water loss (TEWL). Results showed no significant change in the TEWL measurement up to day 10 post‐perfusion (Figure 3L). Together, these comparative data demonstrate that vascular perfusion provides substantial and functionally important advantages over diffusion‐based culture approaches for maintaining the metabolic and structural integrity of full‐thickness tissue over clinically and experimentally relevant timeframes.

FIGURE 3Optimized perfusion maintains tissue viability. (A‐C) Viability of the cells in perfused tissue was analyzed by TUNEL staining (A, C) and quantified (B) (Representative of 5 skin flaps). Scale bar=100 µm (20x objective). (D) Real‐time PCR quantification of HIF 1α, IL‐6, and NF‐κB (*n*=4). (E) Schematic of experimental plan for cell isolation and viability testing analysis from perfused tissue (right panel) and cultured viable cells demonstrated in bright‐field and fluorescent microscopy stained with smooth muscle actin stain. Scale bar=50 µm (20x objective). (F) Schematic of experimental plan for cell isolation and viability testing analysis from non‐perfused control tissues (right panel) and cultured viable cells demonstrated in brightfield and fluorescent microscopy stained with smooth muscle actin stain (red). Scale bar=50 µm (20x objective). (G) Quantification of viable cells isolated from perfused tissue and control non‐perfused tissues is shown in Figure F. Viable cells were counted using a Neubauer chamber and trypan blue stain for dead cells. (H) H&E‐stained skin sections of explant diffusion cultured and perfused skin from the same human donor. Epidermal‐dermal separation is indicated by red arrows. Scale bar=50 µm. (*n*=3) (I and J) Histological section of perfused and control tissues stained for perilipin protein (green). Scale bar=50 µm (20x objective). Fluorescent intensity is quantified and plotted (J) *n*=3. (K) Vascular response to stimulus in the form of vasoconstrictor (catecholamines) and vasodilator (papaverine) in the form of change in pressure and flow rate is measured and plotted at different time points post‐perfusion (*n*=2). (L) TEWL measurement from different time points post‐perfusion (*n*=3). *p* value <0.05=^*^, ns= non‐significant.

**Figure:**
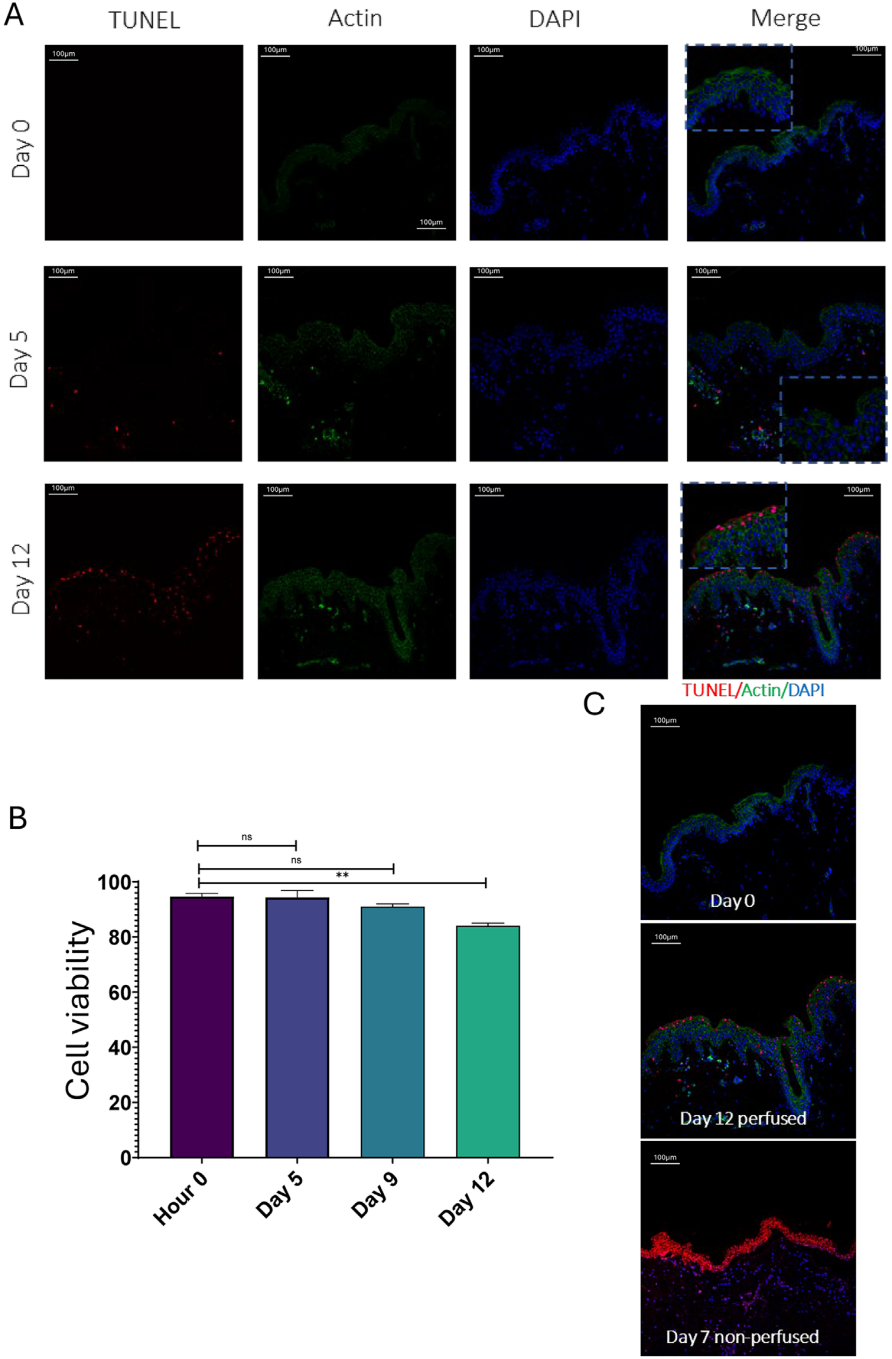

**Figure:**
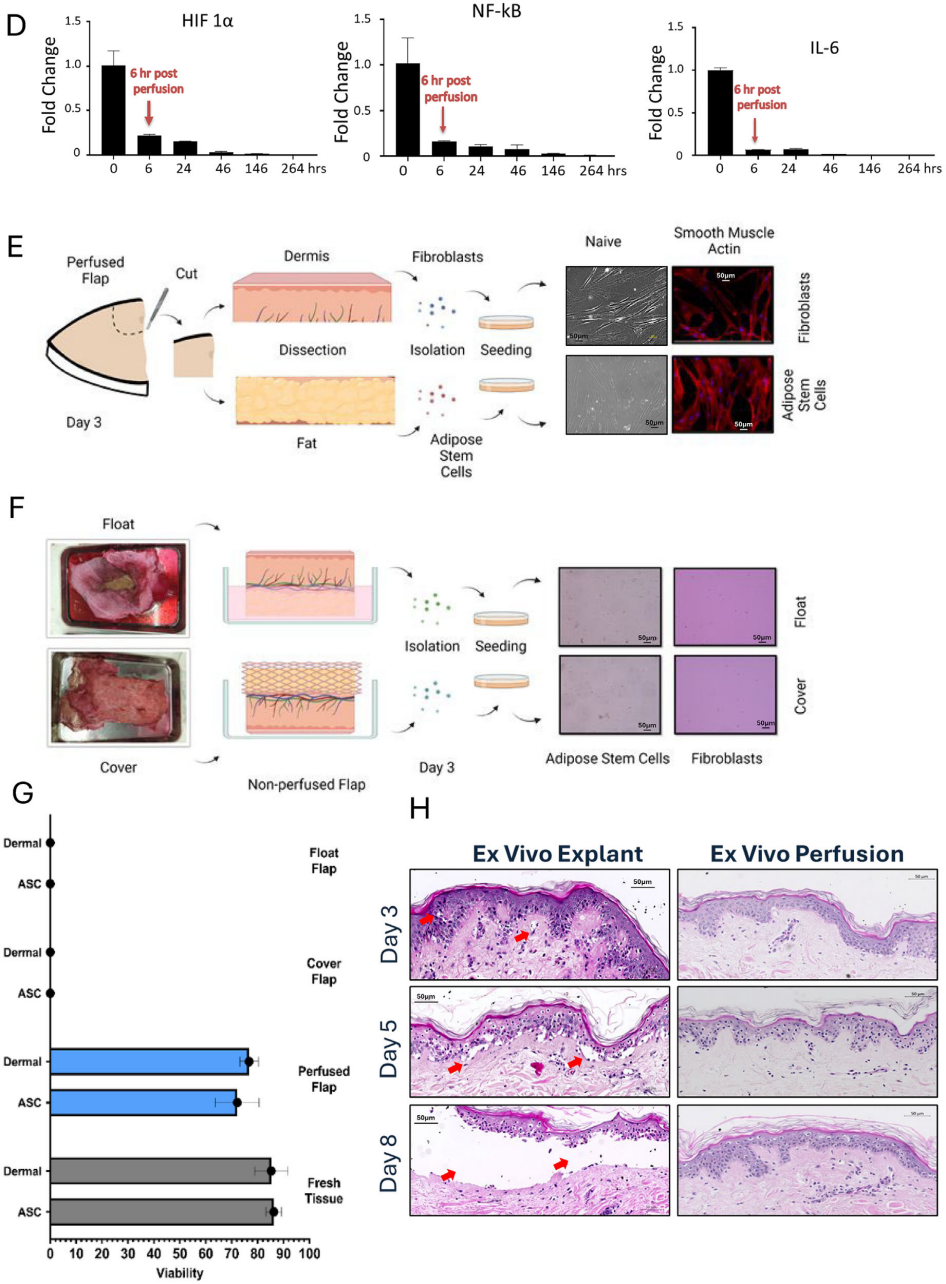

**Figure:**
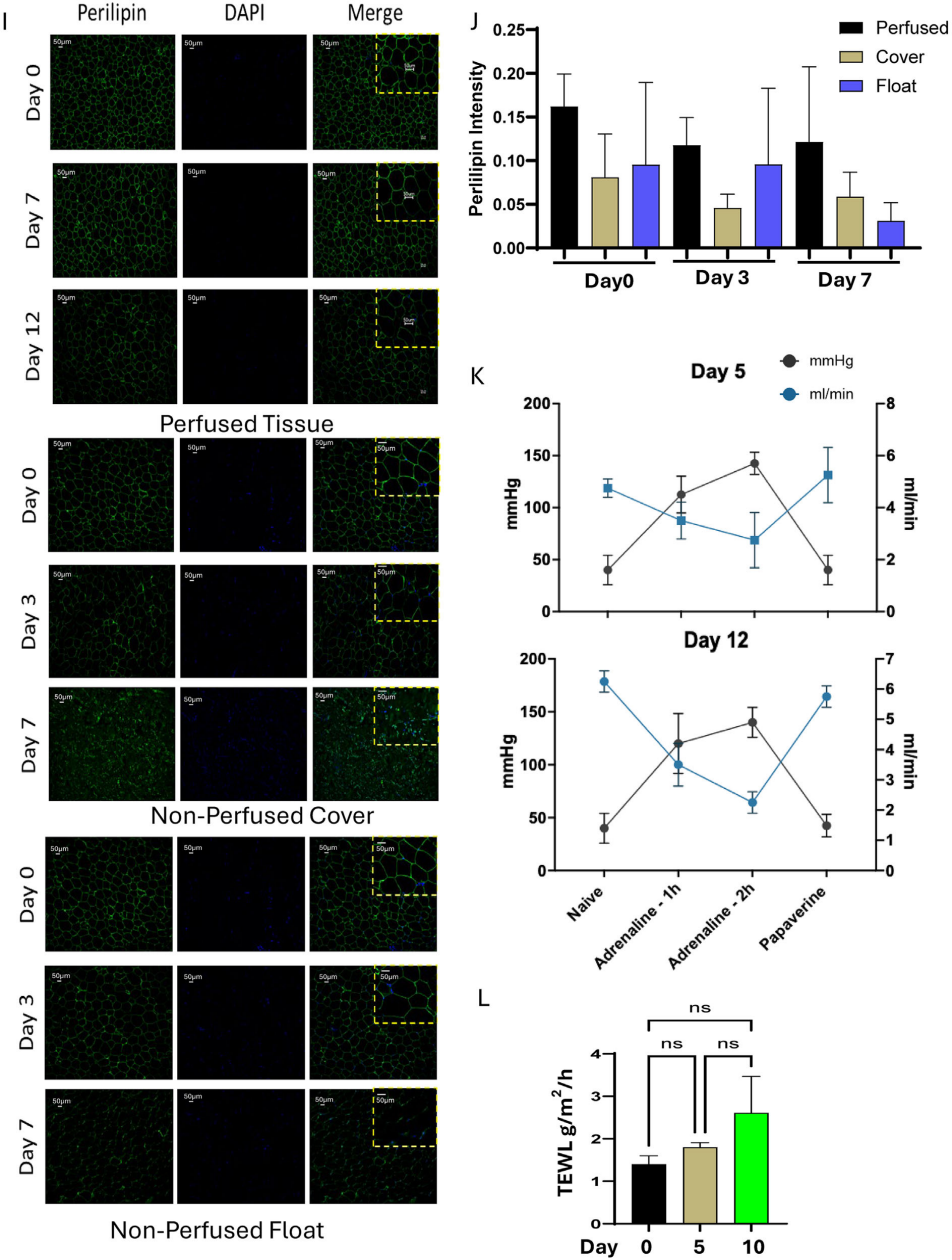

### 3.3 Application of the Human Skin Perfusion System to Understand Radiation‐Induced Skin Injuries

Radiation‐induced skin injuries can result from cancer treatment, radiation therapies or accidental exposure. Existing models for studying radiation‐induced skin injuries lack both anatomical and physiological relevance to humans. In our pursuit of exploring the utility of a human skin perfusion model for investigating radiation‐induced injuries, we subjected human skin to focused radiation doses of 20 and 40 Gy (Figure 4A). Histological examinations of skin sections stained with H&E unveiled several noteworthy observations. We noted the disintegration of the epidermal layer and a noticeable shift of keratinocytes towards the dermal layer, indicative of cellular stress/injury (Figure 4B). Furthermore, an increase in immune cell infiltration assessed by histological analysis, more prominent in the 20 Gy irradiated tissue was observed (Figure 4B). As we have used cell‐free media for perfusion, these cells most likely are tissue‐resident immune cells. By day 12, dermal‐epidermal separation became apparent in the irradiated tissue, while the control skin retained its integrity (Figure 4B). Employing Masson's Trichrome analysis on the skin sections, we observed an augmented staining intensity and a significant reduction in the length of the sub‐papillary and reticular dermal layer spaces, indicating a potential alteration in skin architecture and composition (Figure 4C–E). To investigate the potential cause of the epidermal layer separation, we assessed the health of epidermal layer cells through TUNEL staining at 6, 24 h, and day 6 post‐irradiation. TUNEL staining revealed no significant change in TUNEL positivity between the control and irradiated skin sections at the 6‐ and 24 h time points (data not shown). However, extensive TUNEL positivity was evident by day 6 in irradiated skin (Figure 4F), explaining the later loss of the epidermal layer (Figure 4B).

FIGURE 4Application of the skin perfusion system for studying the response to radiation injury. (A) Presentation of the setup for irradiating a perfused human skin flap. (B) H&E‐stained histological sections of control and irradiated skin at different time points post‐irradiation. Scale bar=100 µm. (C) Masson's Trichrome‐stained sections of control and 20 Gy irradiated skin. Scale bar=100 µm. (D and E) Length between the sub‐papillary and reticular dermal layers is measured and plotted. (F) Viability of cells in irradiated and control tissue stained with TUNEL stain (red), nuclei (Blue) are shown. Scale bar=100 µm (20x objective). (G) Quantitative real‐time PCR was employed to estimate the expression of HIF1α, IL‐1, IL‐6, TNF‐α, NF‐κB, TGFβ, CAT, NQO1, PCNA, SOD1, and RAD54L (*n*=4). *p* value <0.05=^*^, <0.005=^**^, <0.0005=^***^ <0.00005=^****^, ns= nonsignificant.

**Figure:**
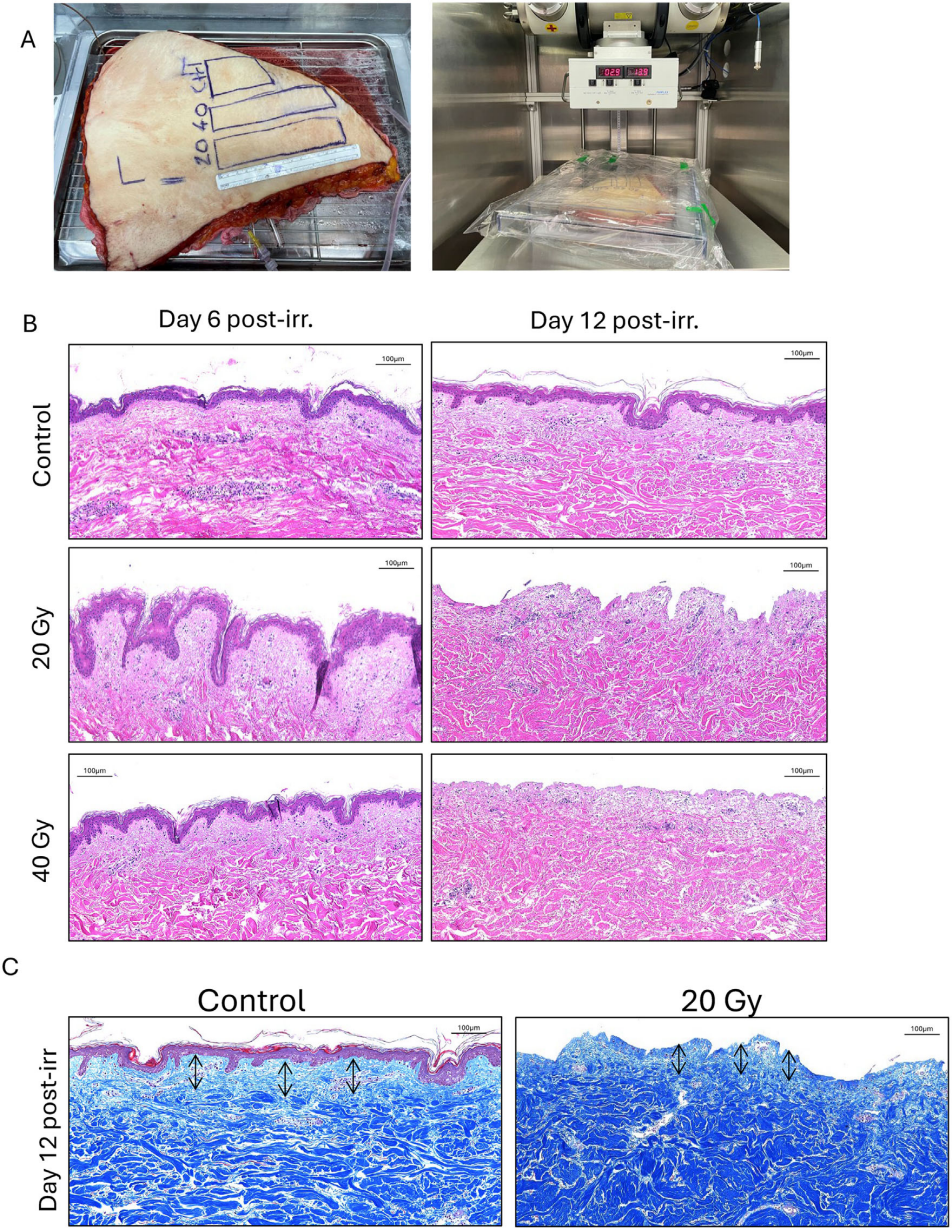

**Figure:**
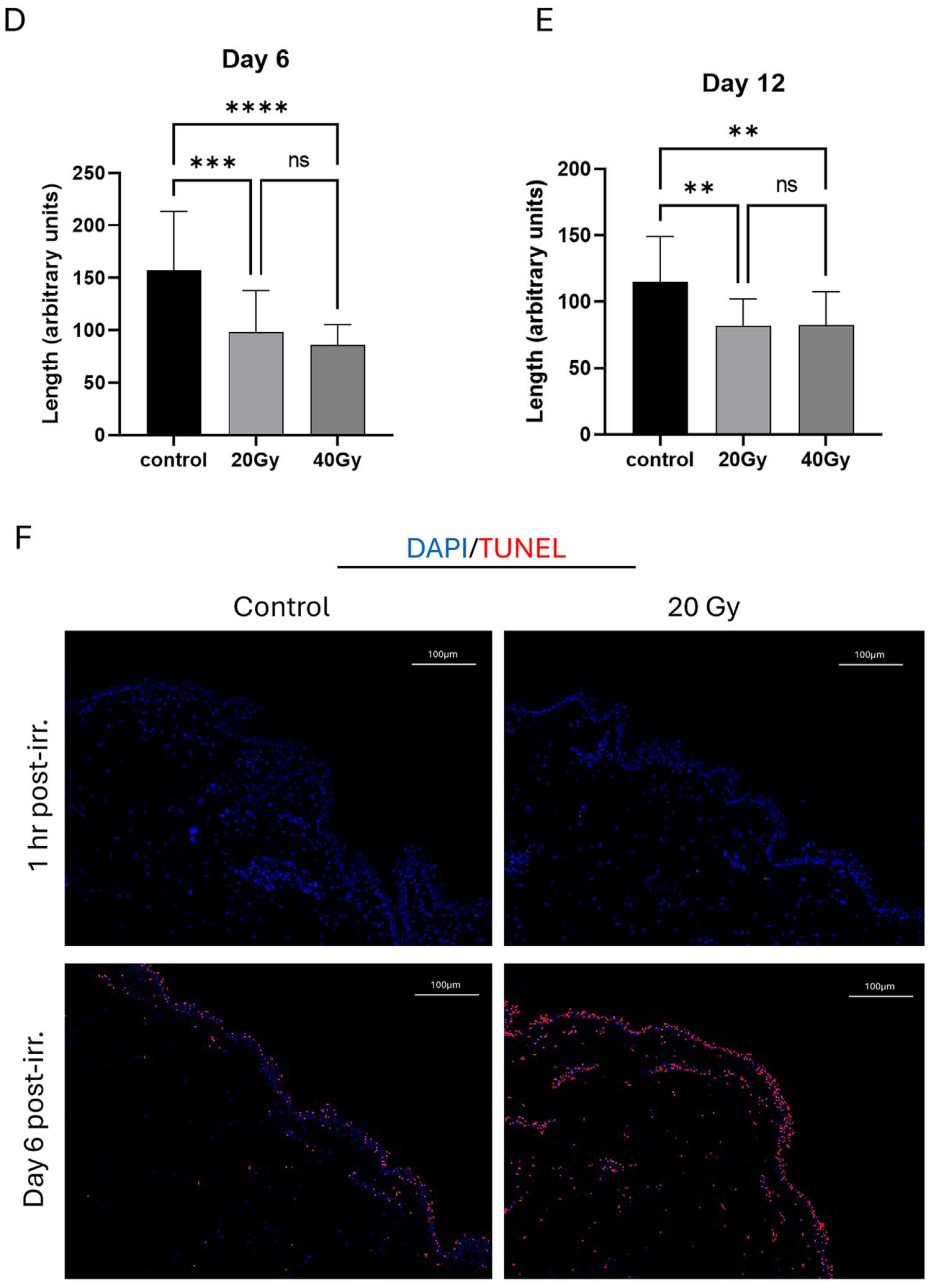

**Figure:**
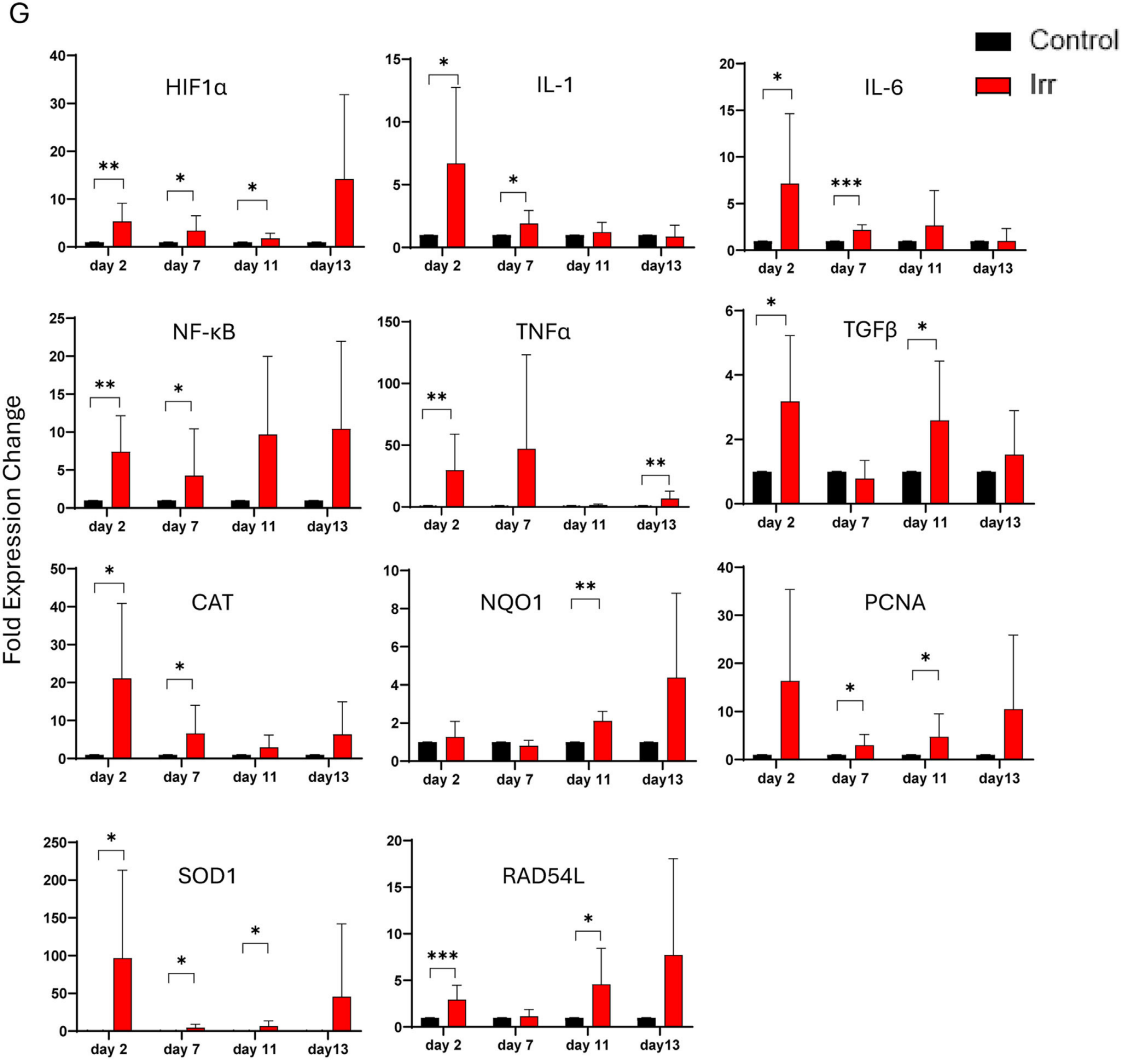

In a prior study, we examined the dynamics of inflammation and fibrosis‐related gene expression in mouse skin at different time points following irradiation [20]. To extend this analysis to human skin, we evaluated the expression of hypoxia marker (HIF1α), inflammatory genes (IL‐1, IL‐6, NF‐κB, TNFα), fibrosis‐related gene (TGF‐β), and damage‐associated genes (Cat, NQO1, PCNA, SOD1, and RAD54L) in human irradiated and control skin from the same donor.

Quantitative Real‐Time PCR results unveiled an increase in hypoxia and inflammation in the irradiated skin tissue (Figure 4G). The upregulation of TGFβ gene expression, a primary driver of the fibrotic response post‐irradiation, commenced within 24 h of radiation exposure and remained elevated (Figure 4G). Genes involved in oxidative defense and DNA repair CAT, PCNA, SOD1, and RAD54L were also upregulated in irradiated skin, indicating induction of possible cell damage (Figure 4G).

Furthermore, we tested the utility of the skin perfusion platform to help us understand the molecular changes occurring in skin post‐irradiation by employing next‐generation bulk RNA sequencing of the skin samples. Principal component analysis of global transcriptomic variation revealed a clear separation along PC1 axes between the control and irradiated samples, indicating that irradiation drives the largest transcriptional difference between groups (Figure 5A). This dimensional reduction technique highlights the global impact of irradiation on gene expression and sets the stage for identifying specific molecular pathways affected. Volcano plot analysis of differentially expressed genes revealed a total of 79 significantly upregulated and 208 down‐regulated genes in irradiated skin compared to control non‐irradiated samples (Figure 5B). The distribution of differentially expressed genes among the individual samples is depicted in the form of a heat map (Figure 5C). Heat‐map data reflect slight transcriptional variation among donors to irradiation, emphasizing the benefits of using experimental models reflecting true variations in population dynamics and responses (Figure 5C). Pathway enrichment was assessed using z‐score‐based activity prediction (Figure 5D). Negative z‐score reflects pathway inhibition, while a positive z‐score indicates activated pathways. Strong inhibition was observed in oxidative and inflammatory pathways, such as RHO GTPases activate NADPH oxidases, detoxification of reactive oxygen species, and production of nitric oxide and reactive oxygen species in macrophages, indicating an intrinsic tissue effort to reduce ROS generation and immune activation. In contrast, positive enrichment of Leukocyte Extravasation Signaling, Elastic Fiber Formation, HIF1α Signaling, IL‐17A Signaling in Fibroblasts, and Collagen Degradation suggests enhanced vascular remodeling, ECM turnover, and tissue repair responses. These results highlight a transcriptional shift from inflammatory and oxidative stress pathways toward regenerative and matrix remodeling programs, consistent with a pro‐healing molecular environment. Gene ontology (GO) enrichment analysis of bulk RNA‐sequencing data revealed predominant regulation of intracellular and metabolic processes (Figure 5E). The most enriched categories included intracellular organelle, cytoplasm, protein binding, and RNA metabolic process, indicating broad transcriptional changes associated with cellular organization, biosynthesis, and macromolecular metabolism. The transcriptional signatures identified in the perfused human skin model are consistent with clinical observations in radiation dermatitis, including activation of HIF1α signaling and leukocyte recruitment pathways (Figure S6). These differentially expressed genes in the RNA sequencing data were verified by quantitative real‐time PCR (Figure 4G). In conclusion, our findings suggest that the human skin perfusion model recapitulates key molecular and histological features of the early pathogenesis of radiation‐induced injuries, supporting its utility as a promising human‐relevant platform for studying radiation injury dynamics and evaluating mitigation strategies.

FIGURE 5Transcriptomic analyses of irradiated and control human skin (*n*=4). (A) Principal component distribution analyses of transcripts from irradiated and control human skin from individual donors. (B) Volcano plots of RNA sequencing data. The x‐axis represents the log_2_ fold change, indicating how much a gene's expression has increased or decreased, while the y‐axis shows the –log_10_(p‐value), reflecting the confidence in those changes. Genes are color‐coded: red for significantly upregulated (79 genes), blue for significantly downregulated (208 genes), gray for inconclusive (850 genes), and black for statistically insignificant (8704 genes). (C) Heat Map analysis of significantly differently expressed genes. (D) Pathway enrichment analyses. (E) Gene Ontology (GO) enrichment analyses.

**Figure:**
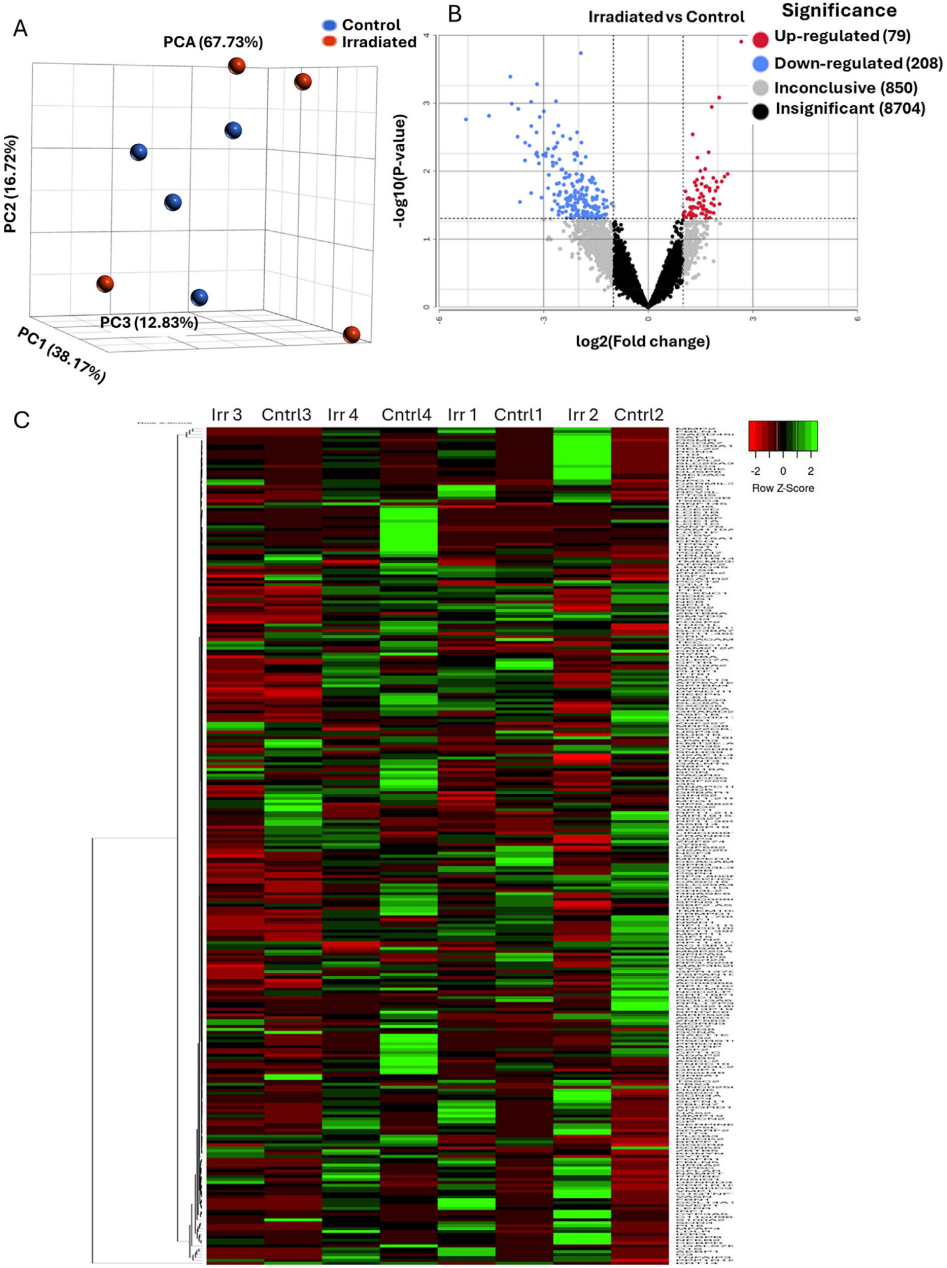

**Figure:**
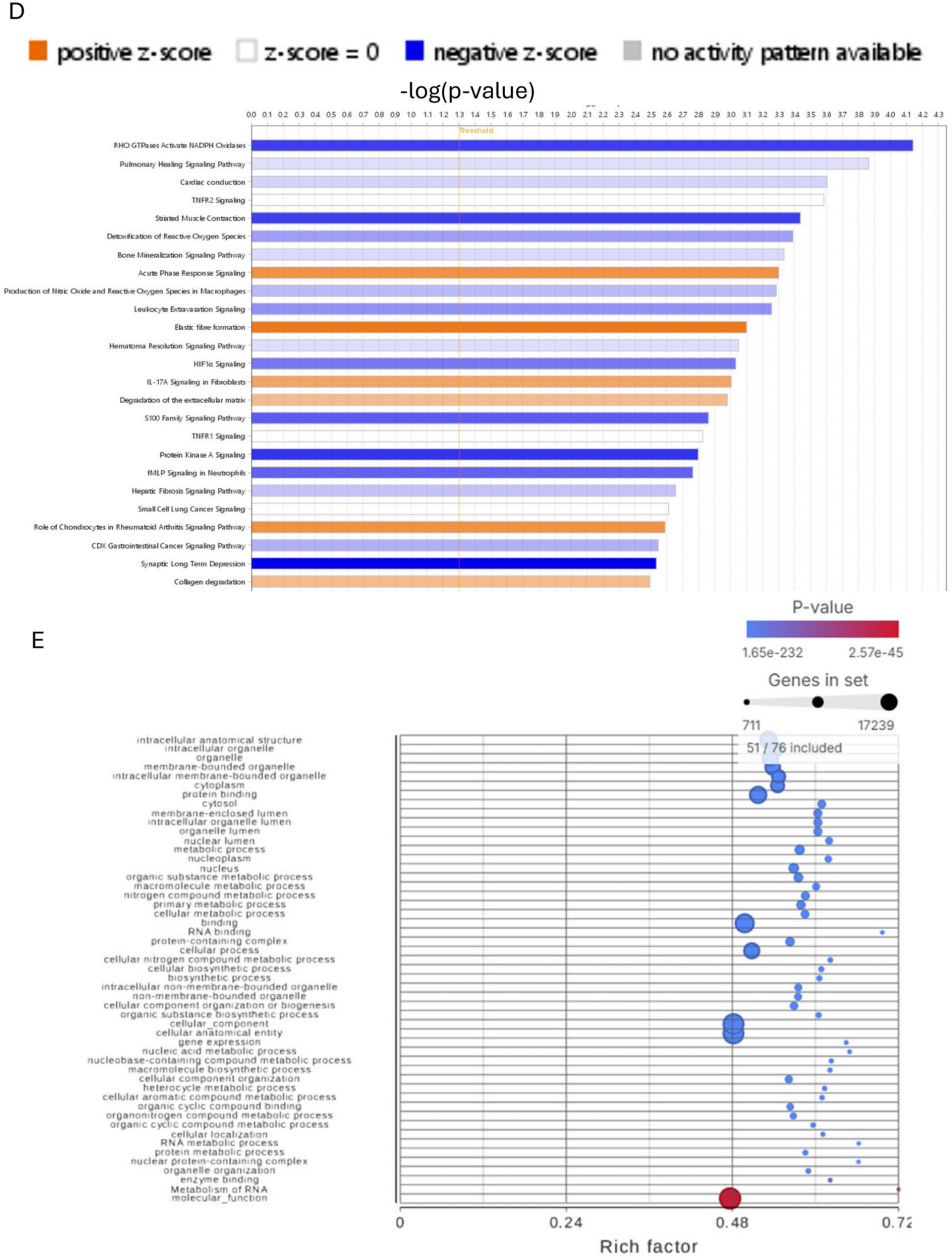

### 3.4 Application of the human Skin Perfusion System to Study Chemical‐induced Skin Injuries

In vitro cell toxicity assays and in vivo animal models have been used to understand the mechanism of vesicant‐induced pathogenesis and to test mitigation strategies. Comparison of vesicant‐induced injuries in different animals and humans revealed differing extent of injury and healing rate, further emphasizing the importance of anatomical architecture and species‐specific tissue physiological and pathological responses [25, 26]. To test whether human skin perfusion system can be utilized to study chemical vesicant induced injuries, we selected nitrogen mustard (NM), a bifunctional analog of sulfur mustard extensively used to study the sulfur induced injuries [27, 28]. These analogs are alkylating agents and target DNA, RNA, proteins, carbohydrates, and lipids [29, 30, 31]. An initial dose of 3 mg/cm^2^ was tested based on the data published in mice and humans [32, 33], but no visible effects were seen (data not shown). Therefore, a higher dose was tested to induce damage. Perfused full‐thickness human skin tissue was treated with a 30 mg/cm^2^ dose of NM in acetone or acetone alone (control) with a pipette, and a pre‐soaked cotton applicator was used to uniformly spread the chemical (Figure 6A, upper panel). *Trans*‐epidermal water loss assessed visually and imaged was prominent as early as 30 min post‐application, indicated by white stars (Figure 6A, middle panel). Biopsies were collected for histology, immunofluorescence, and gene expression analysis. Skin punches are evident in frames from 6 to 200 h. Fluorescein angiography on day 12 confirmed the maintenance of perfusion (Figure 6A, lower panel). H&E‐stained sections revealed pyknosis, ballooning, and focal dermal‐epidermal split (200 hr. post‐treatment) in the NM‐treated skin section (Figure 6B). Perivascular and interstitial lymphocytic inflammation was noted with prominence at 24 h. in NM‐treated skin specimens (Figure 6C).

FIGURE 6Analysis of chemical‐induced skin injuries in perfused human skin. (A) Demonstration of the procedure for inducing chemical (nitrogen mustard) injury in perfused human skin (upper panel). Visible changes in skin post‐nitrogen mustard injury (lower panel), white stars indicate transdermal water loss. (B and C) H&E‐stained histological sections from control and nitrogen mustard‐treated skin. Black arrows indicate inflammatory cells. (B) Scale bar=100 µm (20x objective), lower panel and Scale bar=50 µm (40x objective), upper panel. (C) Scale bar=100 µm (20x objective). (D) (Upper panel) Assessment of cell viability post‐nitrogen mustard treatment using TUNEL stain (Red), Actin (Green), and DAPI (Blue). Scale bar=100 µm (20x objective). (Lower Panel) Quantification and plot of TUNEL‐negative viable cells. (E and F) PARP (E) and caspase 3 (F) immune fluorescence staining. Scale bar=100 µm (20x objective). (G) Quantitative real‐time PCR was employed to estimate the expression of IL‐1, IL‐6, RAD54L, BCL2, CAT, HMGB1, NQO1, and NRF (n=4). *p* value <0.05=^*^, <0.005=^**^, <0.0005=^***^ <0.00005=^****^, ns= non‐significant.

**Figure:**
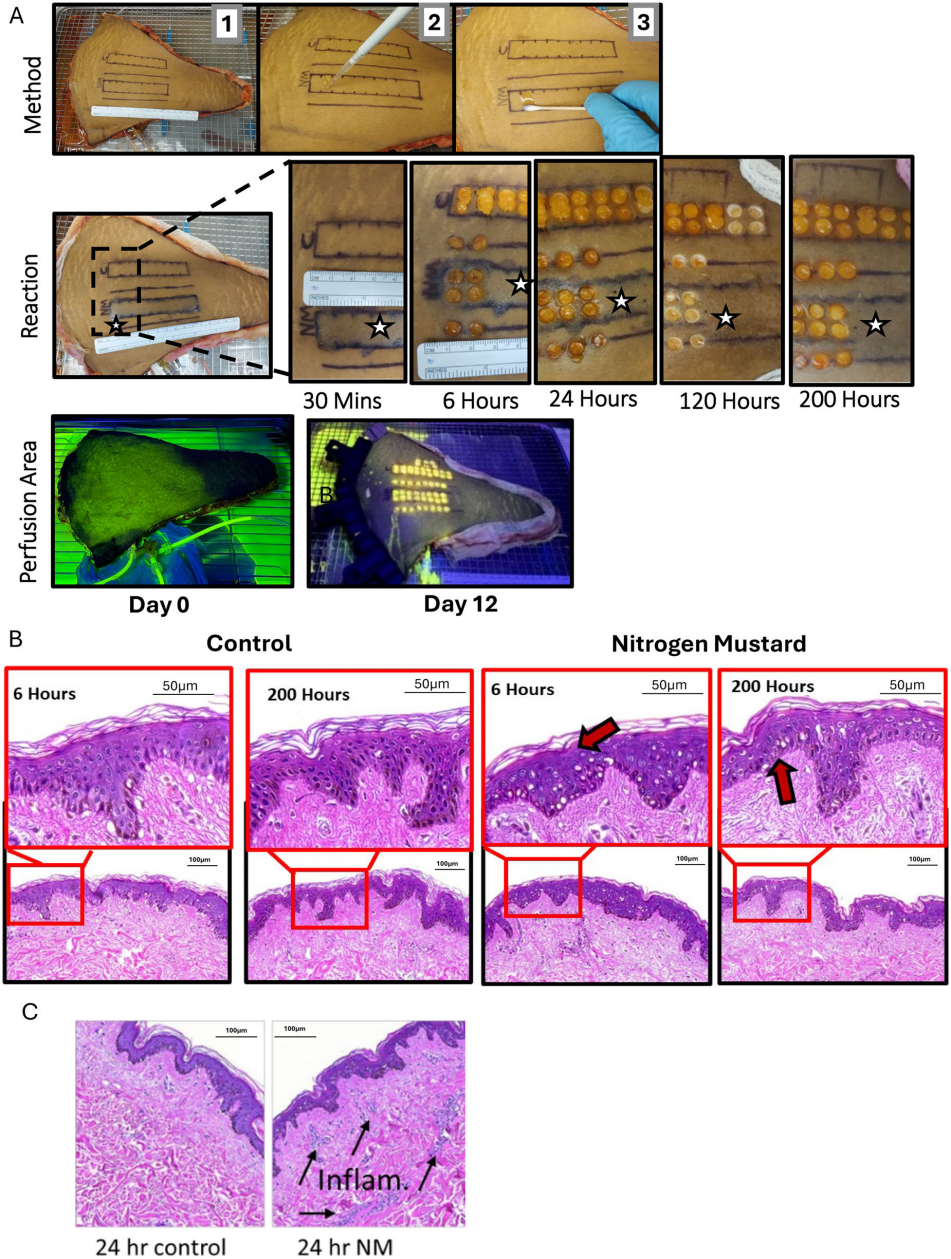

**Figure:**
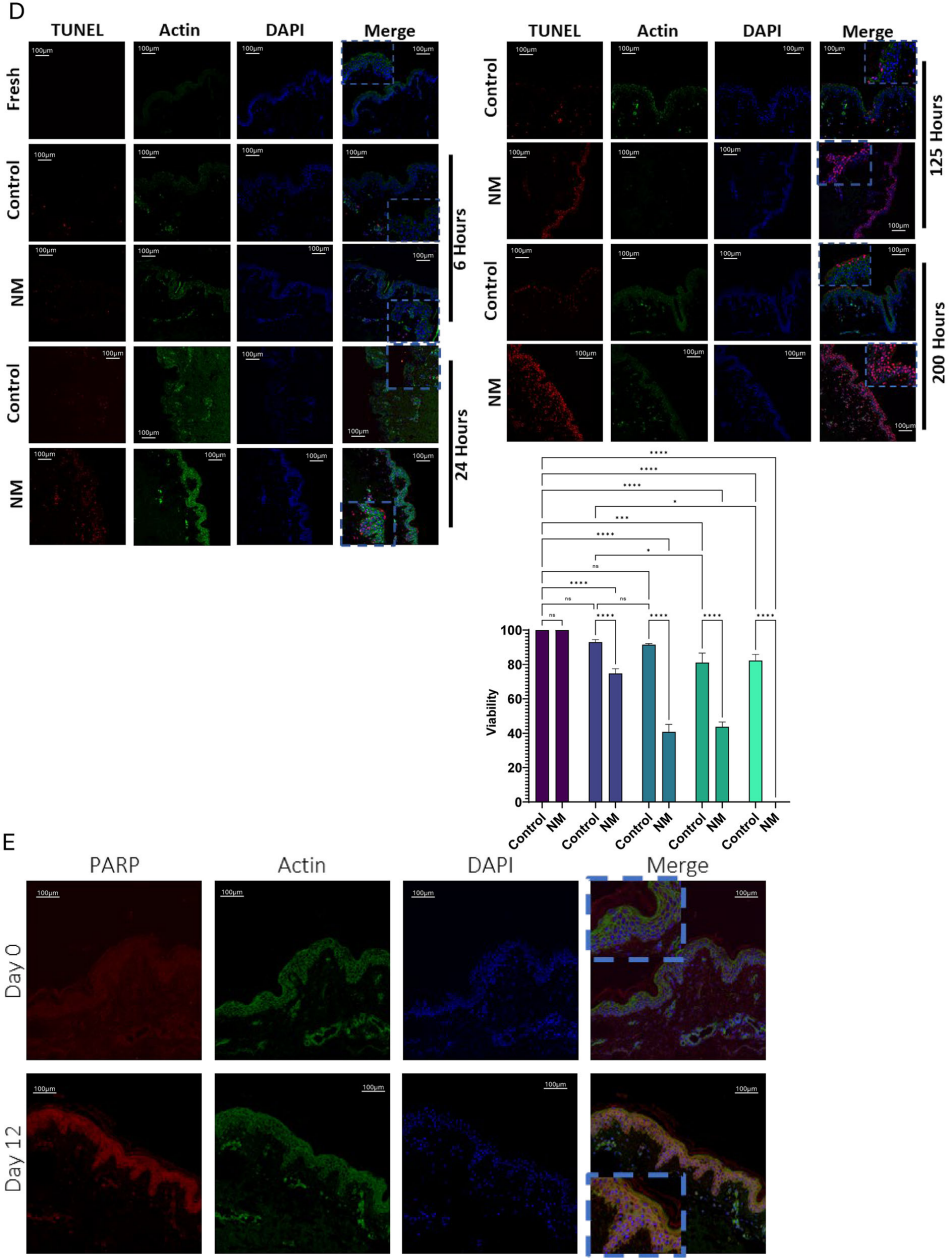

**Figure:**
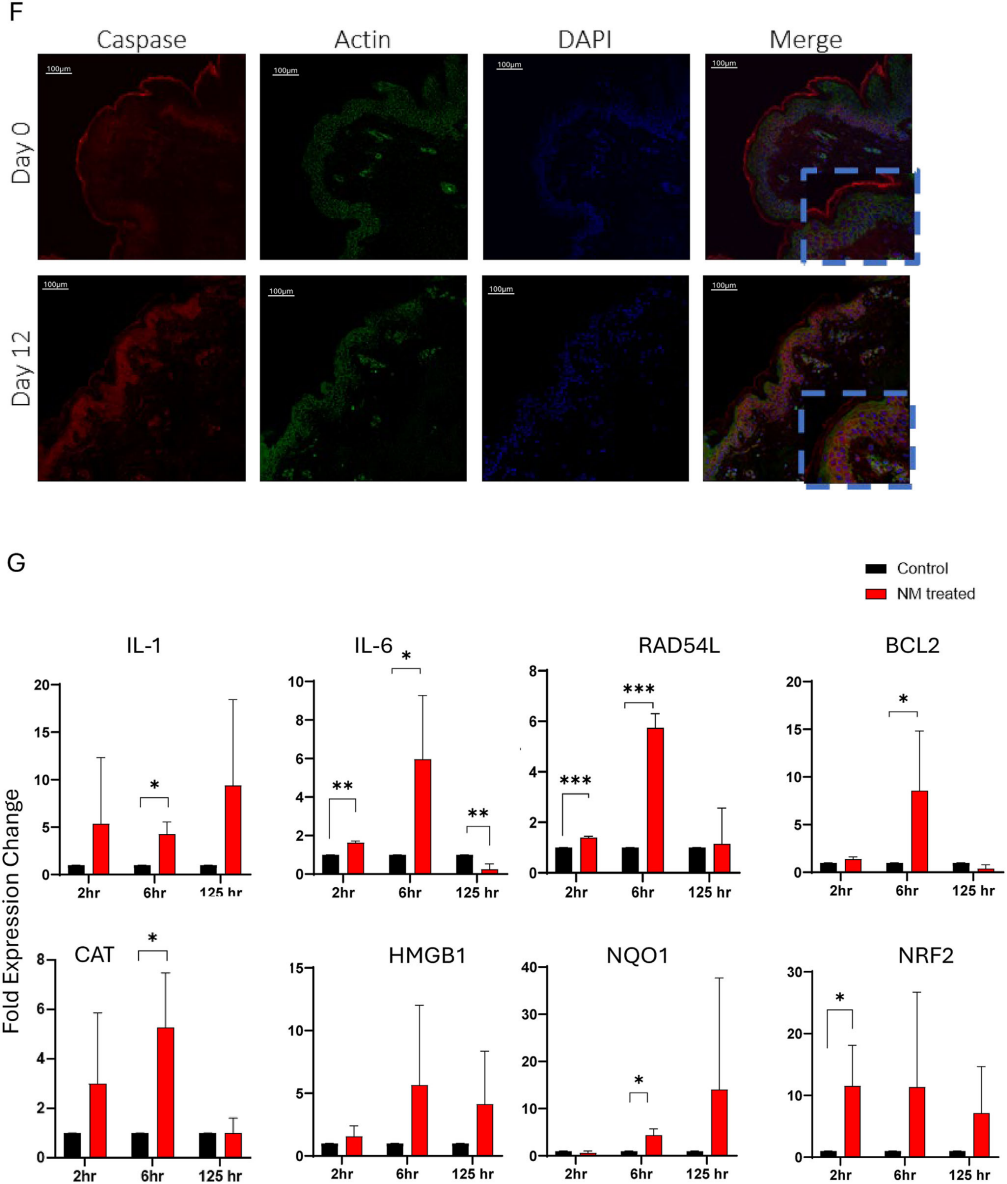

Next, we analyzed the effect of nitrogen mustard treatment on cellular health. TUNEL staining of skin tissue sections at different time points post‐exposure revealed a gradual increase in the magnitude of cell death with time in NM‐treated skin (Figure 6D), and almost all the cells died by 200 h in NM‐treated skin. We also observed TUNEL positivity in the deeper dermal and sub‐dermal layer (Figure S7). Increased PARP activity has been previously reported to be associated with NM‐associated cell death [34, 35, 36]. We analyzed PARP and Caspase 3 expression in NM‐treated skin sections. Confocal microscopic images revealed upregulation of both PARP and Caspase3 activity in the NM‐treated skin compared to control sections (Figures 6E–F and S8). We analyzed a battery of genes indicative of inflammation and apoptotic response (Figure 6G). We observed an increase in inflammation and upregulation of repair‐associated genes in NM treated skin. We conclude that NM treatment induced histological changes and cell death in perfused human skin, which are characteristic of sulfur mustard exposure.

### 3.5 Utilization of the Human Skin Perfusion System to Study Adipose Tissue Biology

**FIGURE 7:**
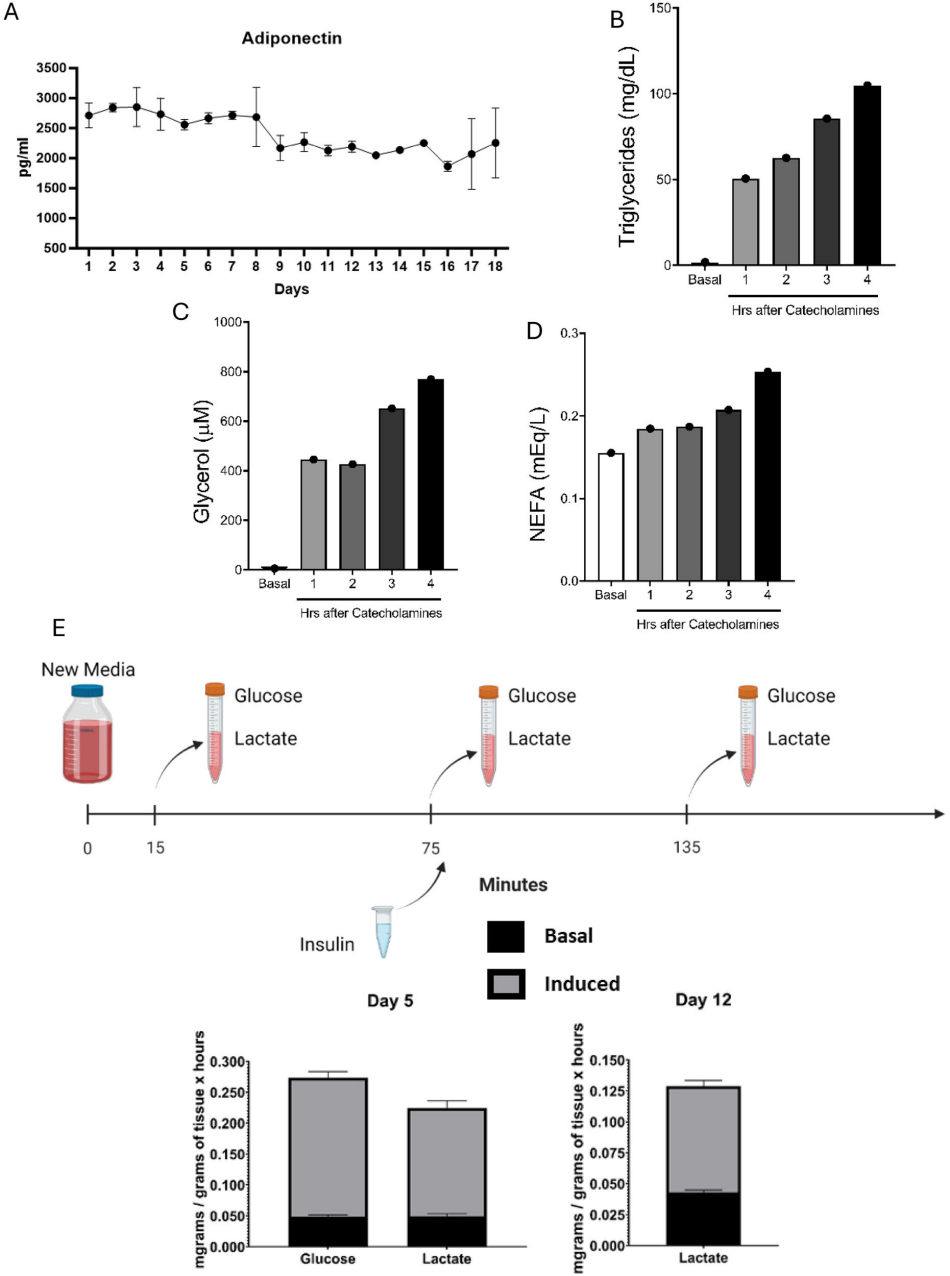
Metabolic functional analyses of adipose tissue. (A) Quantification of adiponectin levels in perfusate by ELISA. (B, C, and D) Quantification of triglycerides (B), NEFA (C), and glycerol (D) in perfusate in response to catecholamine challenge. (E) Schematic of insulin challenge experiment (upper panel), quantification of glucose uptake and lactate production at different time points post perfusion.

Subcutaneous adipose tissue plays an important role in energy homeostasis. Our full‐thickness human skin tissue has the subcutaneous adipose tissue layer intact; therefore, it can be a valuable model to study adipose tissue biology. To analyze the health of adipose tissue in our perfused skin tissue, we analyzed the production of adipose‐associated hormone adiponectin in the perfusion media throughout our perfusion run‐up to 18 days. Results revealed a steady production of adiponectin through the run (Figure 7A).

Next, we tested the metabolic function of adipocytes by measuring their lipolytic response to catecholamine stimulus. Perfusion with catecholamine on day 12 after initiation of perfusion demonstrated a time‐dependent increase in triglycerides, non‐essential fatty acids (NEFA), and glycerol levels in the perfusion media (Figure 7B–D). Adipocytes respond to insulin stimulus by increasing glucose uptake. We challenged the perfused skin tissue by adding insulin to the perfusion media and analyzed the glucose uptake and lactate production on days 5 and 12. Results showed almost a 5 times increase in the glucose consumption by the tissue and lactate production upon insulin induction (Figure 7E). Results shown in Figure 3E demonstrated that ASCs are maintained in perfused skin tissue. Our analyses concluded that the skin perfusion system can be a suitable and novel tool to study questions and manipulations related to adipocytes and ASCs biology in their natural environment.

### 3.6 Human Skin Tissue Perfusion Model Application for Tumor Modeling

Tumor models can be categorized as in vitro, in vivo, and ex vivo models. In vivo tumor models, mainly consisting of patient‐derived xenograft (PDX) and humanized mouse models, have their shortcomings in the form of a lack of human relevance, anatomic differences, genetic diversity, and cost‐effectiveness [37, 38]. We initially applied our novel human skin perfusion model for melanoma modeling. We analyzed the tumor‐forming capabilities of ATCC melanoma cell lines MV3, A375, and patient‐derived cell lines OMM 2.3. To facilitate cell tracking, we transduce the A375 and OMM 2.3 with lentiviruses encoding green fluorescence protein (GFP). We injected 5 × 10^6 cells using a 16‐gauge syringe needle intradermally in the skin on day 1 post perfusion (Figure 8A). The full‐thickness skin tissue was continuously perfused for 14 days. Tissue biopsies were collected on day 14 and fixed in 10% formalin for histological processing. H&E staining of paraffin sections revealed robust tumor formation and neoplastic changes in melanoma cells in the tissue environment (Figure 8B). Examination of H&E‐stained sections further revealed that melanoma cells did not remain confined to the intradermal injection site but demonstrated directed migration along the fascia planes in the hypodermis (Figure 8B), a histological pattern that recapitulates the perivascular and subfascial invasion routes observed clinically in metastatic melanoma and is consistent with the tumor cells exploiting the tissue's natural anatomical planes. This organized invasive behavior, distinct from passive diffusion, represents strong evidence of functional tumor‐tissue crosstalk in our model. Intriguingly, we observed melanin production by the tumor cells inoculated in the skin (Figure 8C). To confirm the identity of the injected cells, we stained the sections of skin injected with OMM2.3‐GFP cells versus control cells. Confocal imaging of the stained slides confirmed the presence of GFP‐positive cells at the site injected with OMM2.3‐GFP cells, while no GFP signal was observed in the sections obtained from the site injected with control tumor cells (Figure 8D). We further confirmed the expression of Sox‐10, a well‐known marker for melanoma cells in melanoma‐injected skin sections. Confocal microscopy results revealed cells positive for Sox‐10 in skin sections injected with melanoma cells and not in control skin (Figure 8E). Proliferation of injected melanoma cells in perfused skin tissue is confirmed by a Ki‐67‐positive signal in the injected cells (Figure 8F).

FIGURE 8Tumor modeling in perfused human skin(A) Inoculation of tumor cells in perfused human skin. (B) H&E staining of human skin biopsies injected with media (control) or melanoma cell lines OMM2.3 and MV3. Scale bar=50 µm (10x objective), left panel; Scale bar=50 µm (20x objective), middle panel; Scale bar=50 µm (40x objective), right panel. (C) H&E‐stained sections of melanoma (MV3) injected skin showing melanin production. Scale bar=50 µm. (D) GFP (green), nuclei DAPI (blue)staining for GFP‐expressing OMM2.3 cells and control cells. Scale bar=100 µm (20x objective). (E) control and MV3 injected sections stained for SOX‐10 (green), actin (red), and nuclei (Blue). Scale bar=100 µm. (F) H&E‐stained section of skin injected with melanoma A375 cells (upper panel) and sections stained with proliferation marker Ki‐67 (green) and DAPI (blue). Scale bar=500 and 20 µm (10x objective). (G) H&E‐stained sections of skin injected with breast cancer cell lines MCF‐7HTB and MDA‐MB‐231. Scale bar=50 µm (10x objective), left panel; Scale bar=50 µm (20x objective), middle panel; Scale bar=50 µm (40x objective), right panel.

**Figure:**
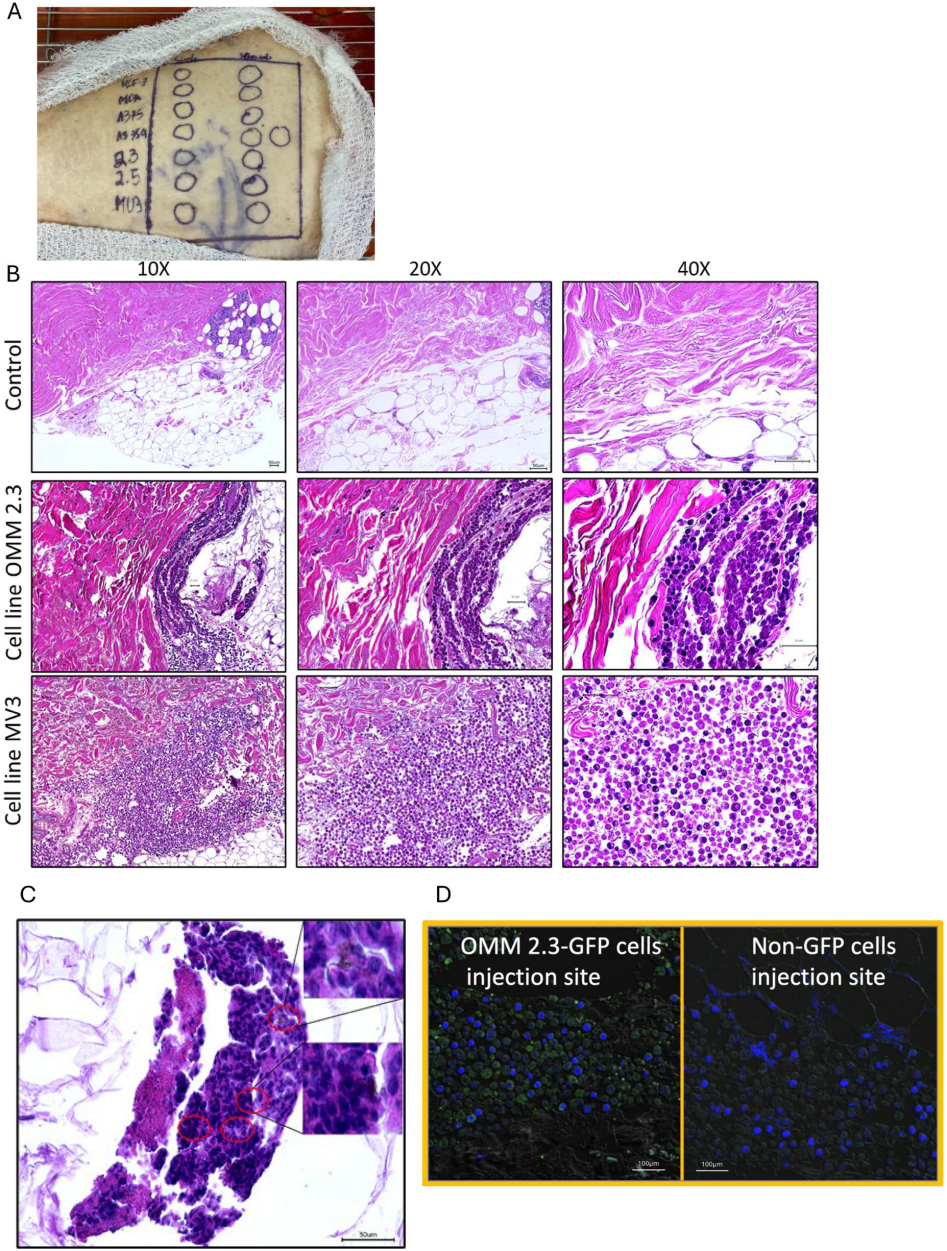

**Figure:**
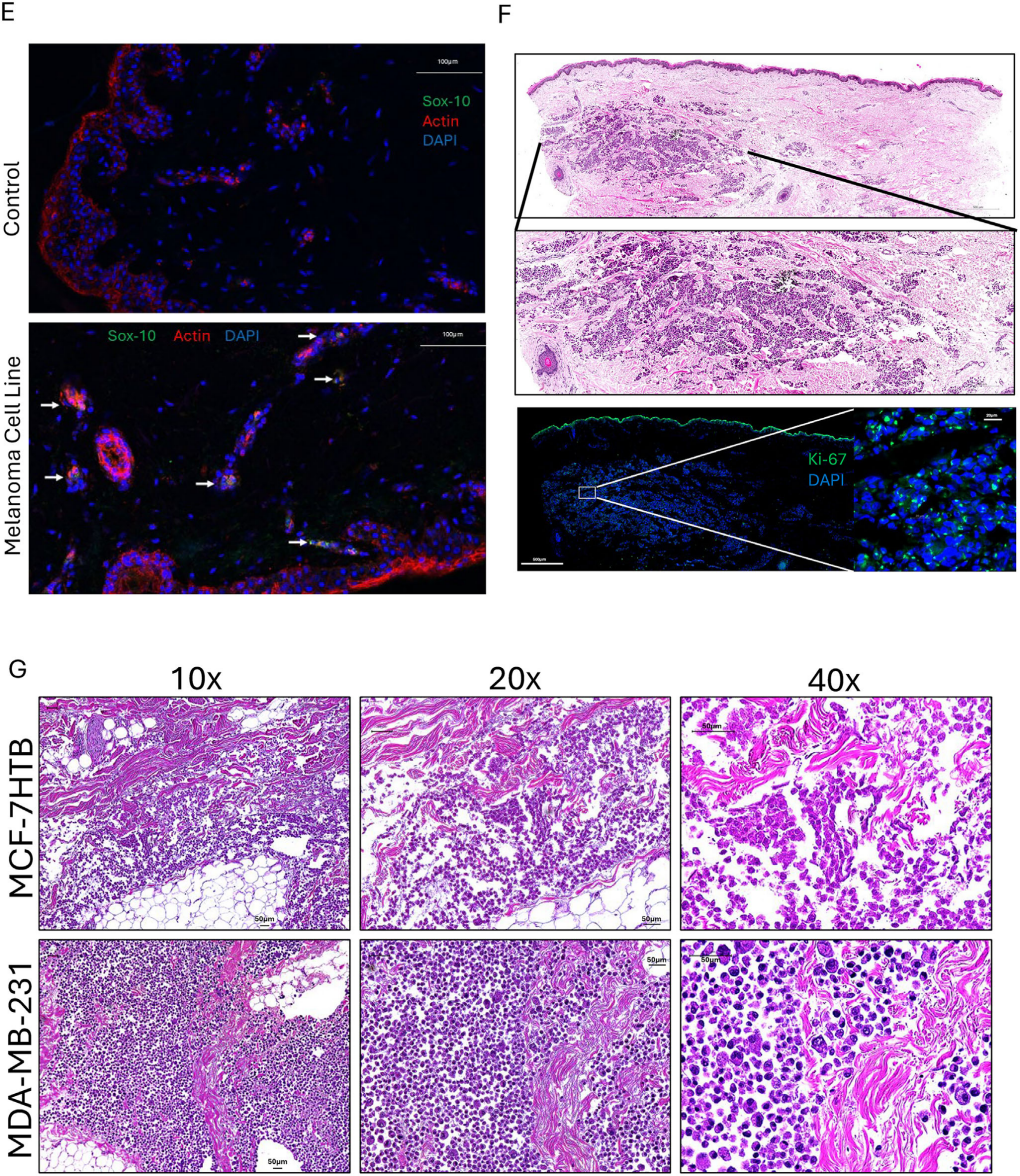

Next, we validated the utilization of our skin model for breast cancer modeling. The rationale for using human skin as a breast cancer model is based on analysis of a breast cancer registry of 1891 patients at the University of Pittsburgh Cancer registry [39] that revealed an incidence rate of 8.3% skin metastasis, having an alarmingly high mortality rate of 7%. These clinical data provide evidence for breast cancer cell migration, homing, and proliferation in skin tissue, adding a strong justification for exploring the use of the skin perfusion platform for breast cancer modeling. In addition, our full‐thickness skin model has an adipose tissue layer intact that provides an adipose‐rich microenvironment like breast tissue for breast cancer cell proliferation. Given the limited availability of breast cancer models that incorporate a native adipose microenvironment and resident immune cells, our platform may offer complementary advantages for studying tumor‐stromal interactions in a human tissue context. Injection of 5 × 10^6 MCF‐7 or MDA‐MB‐231 cells intriguingly resulted in neoplastic cells on day 12 post‐injection in ex *vivo* perfused human skin (Figure 8G). These results provide initial evidence supporting the feasibility of using the human skin perfusion model for tumor engraftment studies, with drug discovery and therapeutic testing applications to be addressed in future work.

## 4 Discussion

Ex vivo models offer assessment of specific effects on cells and surrounding tissue components compared to in vivo models. Although skin ex vivo models have been utilized in various studies, a comprehensive approach to optimizing and assessing skin viability over an extended period, as well as examining the fidelity of skin behavior in response to injury, has been lacking [40]. Through anatomical optimization of vascular access, functional monitoring of perfusion dynamics, and comprehensive molecular and histologic analyses, we demonstrate that the superficial inferior epigastric artery (SIEA) and vein (SIEV) represent the most effective pedicles for perfusion of panniculectomy‐derived human skin, enabling reproducible tissue viability and coverage exceeding 80% of the flap area. This platform maintains vascular tone, metabolic activity, and cellular heterogeneity, and exhibits physiologic responses to stress, injury, and repair stimuli that closely mirror in vivo human tissue biology.

We developed a perfusion bioreactor proficient in sustaining and monitoring optimal perfusion pressure, flow rate, perfusate and tissue temperature, and pH levels, as well as oxygenating the perfusion media and maintaining humidity. Furthermore, we refined the media recipe to minimize tissue edema and control the risk of contamination during extended perfusion runs lasting up to three weeks. Comparative studies on pulsatile and non‐pulsatile perfusion outcomes reveal that pulsatile flow enhances organ flow and oxygenation, whereas non‐pulsatile flow leads to peripheral resistance and an increase in mean arterial pressure [41]. In our perfusion bioreactor, we employed peristaltic pumps to generate pulsatile flow, mimicking the natural vascular environment. A notable feature of human tissue‐based model systems is inter‐donor biological variability, which reflects the true heterogeneity of the human population. Rather than a limitation, this variability is an intrinsic strength of our platform. As illustrated in the transcriptomic heatmap (Figure 5C), individual donor‐level variation in baseline and irradiation‐responsive gene expression is captured and preserved by our system, a capability that is fundamentally absent in inbred rodent models. Systematic analysis of how specific donor variables, such as age and BMI, influence platform‐specific outcomes represents a productive direction for future investigations utilizing this model.

The vascular perfusion approach employed in this study confers several mechanistic advantages over diffusion‐based models that directly enable the biological responses we report. First, oxygen and nutrient delivery via the intact capillary network allows cells in the hypodermis and deep reticular dermis, which lie 3–10 mm below the tissue surface and are beyond the practical diffusion limit for oxygen in tissue (∼200 µm), to maintain aerobic metabolism throughout the experimental period. This is evidenced by the sustained low level of HIF1α expression and the normal glucose‐to‐lactate ratio in perfused tissue at late time points. Second, continuous media recirculation provides efficient clearance of metabolic waste products such as lactate and CO_2_, preventing progressive acidosis that rapidly compromises cell viability in static culture systems. Third, pulsatile intravascular flow maintains physiological mechanobiological stimuli on the vascular endothelium, as confirmed by the robust pharmacological vascular reactivity observed at both Day 5 and Day 12, a property that is absent in microfluidic chips relying on surface‐seeded endothelial monolayers. Fourth, the preserved perivascular niche supports immune cell survival, as shown by the persistence of Langerhans cells, mast cells, macrophages, and dendritic cells, compared to their rapid loss in non‐perfused controls. These mechanistic properties collectively support the extended functional viability, metabolic responsiveness, and tissue‐level injury dynamics observed in our model compared to diffusion‐based ex vivo approaches, though direct head‐to‐head comparisons with advanced microfluidic or organotypic skin systems represent an important direction for future validation. The functional maintenance of perfused human skin tissue in our model is supported by a convergent, multi‐parameter evidence framework spanning five independent biological systems: First, structural integrity: serial H&E and Masson's trichrome histology demonstrates the maintenance of intact stratified epidermal architecture, organized dermal collagen networks, and morphologically healthy adipocyte lobules through Day 15‐20 of perfusion. The preservation of these structures across all tissue compartments confirms that perfusion maintains not only surface viability but the full‐thickness three‐dimensional tissue architecture. Second, metabolic activity: sustained aerobic glucose consumption and lactate production at physiologically meaningful ratios throughout the three‐week perfusion window demonstrate ongoing cellular energetics across the tissue, with the transition from an initially elevated metabolic rate to a stable, lower‐rate steady state consistent with post‐ischemic tissue normalization. Third, cell viability and proliferative potential: TUNEL staining confirms <10% apoptosis in key compartments through Day 9, with the modest increase at Day 12 confined to the outer epidermal layer and reflecting the physiological keratinocyte turnover cycle rather than pathological cell death [40]. Critically, viable ASCs and dermal fibroblasts isolated from perfused tissue demonstrated robust proliferation in cell culture, confirming functional cellular viability at the clonal level.

Fourth, vascular functionality: pharmacological vascular reactivity testing at Day 5 and Day 12 demonstrated a ∼3‐fold pressure increase in response to adrenaline (vasoconstrictor) followed by pressure normalization with papaverine (vasodilator). This response requires intact, biologically competent endothelial cells capable of calcium signaling, receptor‐mediated contractility, and nitric oxide‐mediated relaxation, making it strong evidence of endothelial physiological function in our model. In addition, fluorescein angiography at perfusion initiation and at Day 12 (Figure 6A) demonstrates well‐defined, discrete vascular territories without diffuse extravascular dye leakage or gross extravasation of fluorescein into the interstitium, which would be expected with significant vascular permeability, was not observed. A formal quantitative vascular permeability assay, such as trans‐endothelial electrical resistance and dextran flux, can be adopted for more rigorous validation. Fifth, immune cell preservation: immunofluorescence staining confirms retention of Langerhans cells (langerin+), mast cells (tryptase+), macrophages (CD11b+), and dendritic cells (CD11c+) in perfused tissue. The preservation of this resident innate immune network, which is not present in any 3D organotypic model, is a functionally important property that enables immune‐competent modeling of injury and tumor responses. Together, this five‐tier evidence hierarchy across structural, metabolic, cellular, vascular, and immune parameters provides a comprehensive and convergent case for multi‐system functional maintenance of the perfused skin platform that extends substantially beyond what is captured by metabolic markers alone.

Chronic skin ulcers and burn wounds stand as pivotal wound‐healing challenges, contributing significantly to both morbidity and mortality. Globally, there are reports of 6 million cases of cutaneous diseases and 8 million new burn cases annually [42, 43]. While less frequent, chemical skin injuries also pose substantial risks of mortality and morbidity [44]. Cancer and tumor research represent another crucial field in medicine, with an anticipated 1.8 million new cancer cases expected to be diagnosed in the United States. We demonstrated the impact of various radiation doses on skin tissue histology for up to 12 days, concurrently analyzing the expression of genes related to inflammation and fibrosis in the skin. Although the current lifespan of our skin perfusion model may not be sufficient for studying late side effects of injury, it offers the possibility to conduct the longest analyses of outcomes in an ex vivo injury approach, focusing on early to intermediate effects. We successfully monitor dermal‐epidermal separation and observe an increase in the intensity of Masson's Trichrome stain post 20 or 40 Gy irradiation.

Moreover, our system provides an opportunity to test therapeutics in the early and intermediate phases of injury. Previous studies using human skin biopsies submerged in a cell culture dish and exposed to 0.5 and 5 Gy did not observe any upregulation of TGFβ [45]. In our system, utilizing 20 and 40 Gy doses, we observed upregulation of TGFβ at different time points, indicating a dynamic regulation of gene expression in human skin post‐irradiation. RNA‐seq pathway enrichment revealed suppression of oxidative and ROS‐generating networks and activation of HIF1α, IL‐17A, and collagen turnover pathways. The transcriptional landscape we observe in our perfused model closely mirrors the molecular pathogenesis of clinical radiation dermatitis, which can be broadly divided into acute and chronic phases. In the acute phase (hours to days post‐exposure), radiation triggers DNA double‐strand breaks, rapid activation of NF‐κB signaling, and upregulation of pro‐inflammatory cytokines, including IL‐1, IL‐6, and TNF‐α [46]. Consistent with this, our qPCR data (Figure 4G) show early induction of HIF1α and NF‐κB within 6–24 h post‐irradiation, and our RNA‐seq pathway analysis identifies significant activation of Leukocyte Extravasation Signaling and HIF1α pathways (Figure 5D). Clinically, this acute phase manifests as erythema, edema, and desquamation, driven in part by keratinocyte apoptosis, a process reflected in our TUNEL data showing extensive positivity by Day 6 post‐irradiation (Figure 4F).

In the subacute to chronic phase (days to weeks), the dominant response shifts toward TGF‐β‐mediated fibrotic remodeling, characterized by progressive collagen deposition, vascular remodeling, and loss of skin elasticity [45, 46]. Upregulation of TGF‐β beginning within 24 h post‐irradiation and its sustained elevation (Figure 4G), combined with RNA‐seq‐identified activation of IL‐17A Signaling in Fibroblasts, Elastic Fiber Formation, and Collagen Degradation pathways (Figure 5D), recapitulate this fibrotic transition at the transcriptional level. Notably, RNA‐seq also identified strong inhibition of oxidative and ROS‐generating networks (Figure 5D), consistent with the documented intrinsic antioxidant response of irradiated keratinocytes and fibroblasts, a molecular adaptation also reflected in the upregulation of CAT and SOD1 (Figure 4G). Together, the concordance between our model's transcriptional output and the known molecular pathogenesis of clinical radiation dermatitis supports the biological relevance of this platform for studying radiation injury and for future preclinical evaluation of radioprotective and regenerative interventions. Further testing with various radiation doses and time points will enhance our understanding of the dynamics of radiation injury and aid in the improved design of therapeutic interventions. In our system, perfused human skin exposed to nitrogen mustard exhibited ballooning of the epithelium, a characteristic feature associated with nitrogen mustard injury [32]. Additionally, we observed a gradual increase in cell death, as confirmed by TUNEL staining.

Adipose tissue not only provides structural support for blood vessels but also serves as an essential component in understanding metabolic changes within the tissue. Moreover, its presence in the perfusion system facilitates the evaluation of adipose tissue‐resident cells' responses to stimuli like NM or irradiations, offering valuable insights into pathologies centered around adipose tissue and enhancing the model's relevance for mechanistic studies and drug development. Thus, the adipose tissue's contribution extends beyond mere structural support, significantly impacting the comprehensiveness and applicability of the experimental findings demonstrated by the response of adipocytes within the perfused tissue to catecholamine stimuli by inducing lipolysis. Furthermore, these adipocytes demonstrated responsiveness to insulin challenges, exhibiting increased glucose uptake and lactate production. The validation of these metabolic responses by adipose tissue‐resident cells opens new avenues for ex vivo manipulation in mechanistic studies and drug development, particularly for addressing pathologies centered around adipose tissue. Our results highlight a loss of perilipin expression by day 3 in skin tissue kept floating in culture media, while perfused tissue maintained perilipin expression for up to 12 days and beyond. Additionally, our perfusion approach led to a significant downregulation of inflammation and hypoxia. The perfused human skin system provides a unique opportunity for ex vivo mechanistic studies and conditional manipulations.

We conducted additional tests to evaluate the capacity of our perfused skin to host and incorporate melanoma and breast cancer cells. Notably, our perfused tissue harbors resident immune cells, and we hypothesize that a fraction of injected tumor cells evade resident immune surveillance to successfully engraft and proliferate within the skin microenvironment, consistent with the known mechanisms of immune escape in melanoma and breast cancer. The relevance of studying breast cancer in skin can be drawn by the fact that analysis of 707 breast cancer patients revealed 30% of the patients had skin metastases, and 24% showed skin metastases as the first sign of extranodal disease [47]. Almost 70% of the patients with skin metastases died within 30 months of diagnosis, indicative of a poor prognosis associated with skin metastases. Further extension of the skin tissue life ex vivo and efforts to integrate immune cells in the perfusion media will enhance the relevance of the skin perfusion system as the model of choice for skin‐related cancer research and therapeutics development.

The current tumor engraftment data provides an initial proof‐of‐concept demonstration of cell survival, active proliferation, and histological evidence of tissue‐invasive behavior within the perfused skin microenvironment, representing necessary first steps toward establishing this platform as a tumor modeling tool. The logical and imminent extension of this work is the assessment of anti‐tumor drug responses in a human tissue context, which represents a compelling and currently unmet need in oncology drug discovery. Our model offers several unique advantages for this application: (1) intravascular drug delivery has the potential to enable pharmacologically relevant drug exposure through the native vasculature rather than simple topical application; (2) the presence of resident immune cells may permit future evaluation of immunomodulatory responses in a partially immune‐competent human tissue context, including assessment of checkpoint blockade therapies once appropriate experimental protocols are established; and (3) the intact adipose microenvironment provides an adipose‐rich stromal context particularly relevant for breast cancer and melanoma drug testing. Our laboratory is currently conducting experiments evaluating the response of melanoma tumors to BRAF inhibitor (vemurafenib) treatment in this model, and we anticipate these results will be presented in a follow‐up study. The data presented here, demonstrating Ki‐67^+^ tumor cell proliferation and histological patterns consistent with tissue‐invasive migration, provide encouraging preliminary support for pursuing next‐generation pharmacological studies in this model. Despite several advantages of the ex vivo full‐thickness human skin perfusion system, this system has several limitations. The major ones include technical complexities around the surgical preparation of tissue and the operation of the perfusion bioreactor, accessibility to skin tissue, limited life of the tissue, lack of circulatory immune cells, lack of nerve signals, and lack of lymphatic function. Because of the lack of blood in our perfusion system, we can only observe regenerative changes in this model. However, in future models, we will be looking into the possibility of adding blood products, which would enable us to see wound closure changes on the flap. We have tested both male and female tissues with similar physiological outcomes, but since female tissues are more readily available, we presented the data from female tissues in this study. An additional limitation of the current model is its anatomical specificity: our protocol was optimized for abdominal fasciocutaneous flaps, which offer the highly favorable SIEA/SIEV pedicle geometry that supports large‐territory perfusion coverage. Adaptation of this approach to other anatomical sites will require identification of appropriate perforator pedicles of sufficient caliber and territory. Body regions yielding skin as reconstructive surgical waste, such as the thigh (anterolateral thigh flap donor sites), back (latissimus dorsi flap donor sites), and breast reduction tissue, represent promising candidates for future adaptation, as they are associated with identifiable perforating vessels and are commonly available from elective reconstructive procedures. The technical requirements for successful adaptation include perforator diameter ≥ 1 mm for reliable cannulation, adequate pedicle length for bioreactor integration, and sufficient territory for multi‐application experimental designs. Our laboratory is actively exploring these extensions to broaden the anatomical and clinical applicability of this platform. Our lab is continuously working to address these limitations and enable further adaptation of this system as a model of choice for studying skin pathologies and therapeutics development. Formal quantification of epidermal barrier function by *trans*‐epidermal water loss assays represents an important parameter for future standardized viability characterization of this platform, and we have incorporated TEWL measurements in our current validation protocol.

The tumor modeling experiments presented in this study are intentionally positioned as proof‐of‐concept and feasibility work. The current data demonstrates tumor cell engraftment, histological evidence of proliferation (Ki‐67 staining), and fascial plane migration patterns consistent with invasive behavior, but do not yet constitute robust tumor microenvironment modeling. Specifically, longitudinal quantitative tumor growth kinetics, vascular interaction analysis, immune cell‐tumor crosstalk, and therapeutic drug response experiments have not been performed and represent essential next steps before this platform can be considered a validated oncology research tool. Furthermore, direct experimental comparison of tumor behavior in our perfused model against established systems such as patient‐derived xenografts (PDX), 3D tumor spheroid cultures, or humanized mouse models has not been conducted in the present study. We acknowledge these as important limitations of the current work and are actively pursuing these studies in our laboratory. The use of human tissue discarded as surgical waste to obtain impactful data for developing new treatment strategies has enormous ethical value. Additionally, ethical benefits for using such systems include a lower need to use animals in research. The main ethical consideration for the use of human tissues is to protect the identity and privacy of the donor by de‐identifying the tissue samples.

## Author Contributions

**Y. S**.: Collection and assembly of data, data analysis, and interpretation, manuscript writing. **H. M**.: Collection of data. **A. R. D. R. H**.: Collection of data, manuscript writing. **N. M**.: Collection of data, manuscript writing. **E. B**.: Collection of data, manuscript writing. **D. U. R**.: Collection of data, manuscript writing. **J. A. A**.: Collection of data. **K. S. Y**.: Collection of data. **A. A**.: Collection of data. **G. V. N. K**.: Collection of data. **M. L. S**.: Conception and design. **J. A. G**.: Conception. **J. P. R**.: Conception. **A. E**.: Conception and design, data analysis and interpretation, manuscript writing, and final approval.

## Funding

This study was supported by NIH/NIAID grant 5R21AI153971‐02, 1R34AR083572‐01, 5R21AI153971‐02, and 5U01AI195522‐02 to A.E.

## Conflicts of Interest

The authors declare no conflicts of interest.

## Supporting information




**Supporting File**: adhm71445‐sup‐0001‐SuppMat.pptx.

## Data Availability

The data that support the findings of this study are available from the corresponding author upon reasonable request.
